# Characterization of five complete *Cyrtodactylus* mitogenome structures reveals low structural diversity and conservation of repeated sequences in the lineage

**DOI:** 10.7717/peerj.6121

**Published:** 2018-12-13

**Authors:** Prapatsorn Areesirisuk, Narongrit Muangmai, Kirati Kunya, Worapong Singchat, Siwapech Sillapaprayoon, Sorravis Lapbenjakul, Watcharaporn Thapana, Attachai Kantachumpoo, Sudarath Baicharoen, Budsaba Rerkamnuaychoke, Surin Peyachoknagul, Kyudong Han, Kornsorn Srikulnath

**Affiliations:** 1 Laboratory of Animal Cytogenetics and Comparative Genomics (ACCG), Department of Genetics, Faculty of Science, Kasetsart University, Bangkok, Thailand; 2 Human Genetic Laboratory, Department of Pathology, Faculty of Medicine Ramathibodi Hospital, Mahidol University, Bangkok, Thailand; 3 Animal Breeding and Genetics Consortium of Kasetsart University (ABG-KU), Kasetsart University, Bangkok, Thailand; 4 Department of Fishery Biology, Faculty of Fisheries, Kasetsart University, Bangkok, Thailand; 5 Nakhon Ratchasima Zoo, Nakhon Ratchasima, Thailand; 6 Center for Advanced Studies in Tropical Natural Resources, National Research University-Kasetsart University (CASTNAR, NRU-KU, Thailand), Kasetsart University, Bangkok, Thailand; 7 Bureau of Conservation and Research, Zoological Park Organization under the Royal Patronage of His Majesty the King, Bangkok, Thailand; 8 Department of Biology, Faculty of Science, Naresuan University, Phitsanulok, Thailand; 9 Department of Nanobiomedical Science & BK21 PLUS NBM Global Research Center for Regenerative Medicine, Dankook University, Cheonan, Republic of Korea; 10 Center of Excellence on Agricultural Biotechnology: (AG-BIO/PERDO-CHE), Bangkok, Thailand

**Keywords:** Mitogenome, Control region, Repeated sequence, Mitochondrial gene rearrangement, mtDNA

## Abstract

Mitochondrial genomes (mitogenomes) of five *Cyrtodactylus* were determined. Their compositions and structures were similar to most of the available gecko lizard mitogenomes as 13 protein-coding, two rRNA and 22 tRNA genes. The non-coding control region (CR) of almost all *Cyrtodactylus* mitogenome structures contained a repeated sequence named the 75-bp box family, except for *C. auribalteatus* which contained the 225-bp box. Sequence similarities indicated that the 225-bp box resulted from the duplication event of 75-bp boxes, followed by homogenization and fixation in *C. auribalteatus*. The 75-bp box family was found in most gecko lizards with high conservation (55–75% similarities) and could form secondary structures, suggesting that this repeated sequence family played an important role under selective pressure and might involve mitogenome replication and the likelihood of rearrangements in CR. The 75-bp box family was acquired in the common ancestral genome of the gecko lizard, evolving gradually through each lineage by independent nucleotide mutation. Comparison of gecko lizard mitogenomes revealed low structural diversity with at least six types of mitochondrial gene rearrangements. *Cyrtodactylus* mitogenome structure showed the same gene rearrangement as found in most gecko lizards. Advanced mitogenome information will enable a better understanding of structure evolution mechanisms.

## Introduction

Vertebrate mitochondrial genomes (mitogenomes) are double-stranded circular DNAs typically 16–17 kb in size that encode two rRNA genes, 22 tRNA genes, and 13 protein-coding genes. They also possess non-coding control regions (CRs) which contain signals for the initiation of replication and transcription (Boore, 1999; Fernández-Silva, Enriquez & Montoya, 2003). Mitogenomes are maternally inherited as haploid genomes with multiple copy numbers in a cell. Compared with nuclear DNA, mitogenome gene content is likely conserved with very low levels of recombination, while mitogenome sequences evolved rapidly (Elson & Lightowlers, 2006; Gissi, Iannelli & Pesole, 2008). These features are very useful tools for the resolution of taxonomic controversies (Srikulnath et al., 2012; Sun, Cheng & Zhang, 2015; Supikamolseni et al., 2015; Laopichienpong et al., 2016; Thongtam na Ayudhaya et al., 2017; Prakhongcheep et al., 2018). Simultaneously, nucleotide sequences of the CR containing repeated sequences known as variable number of tandem repeat (VNTR), show high mutation rate which is advantageous for population and phylogeographic studies (Wang, Zhou & Nie, 2011; Lapbenjakul et al., 2017). Although mitogenome organization is likely conserved in vertebrates (Pereira, 2000), mitochondrial gene rearrangements have been found in several groups (Macey et al., 1997; Mauro et al., 2006; Kurabayashi et al., 2008). The majority of these rearrangements result from gene shuffling (such as tRNA genes), translocations and/or duplications of genes, and loss of gene or genome segments (Boore, 1999; Amer & Kumazawa, 2008; Metallinou et al., 2012; Kumazawa et al., 2014). This variation of rearrangements reflects the different functional and evolutionary constraints among taxa (Boore, 1999; Gissi, Iannelli & Pesole, 2008; Pereira et al., 2008; Bernt & Middendorf, 2011).

Recently, the understanding of mitogenomes in squamate reptiles has increased, and technical advances in sequencing have led to rapid accumulation of complete mitogenome sequences (Kumazawa et al., 2014). However, only 25 species have been sequenced from the infraorder of squamate reptiles, Gekkota, which contains more than 1,200 species (Uetz & Hošek, 2018). Approximately 20% of gecko lizard mitogenomes contain various gene rearrangements including tandem duplication of the gene block and loss and reassignment of tRNA genes which were not very common in vertebrate mitogenomes (Fujita, Boore & Moritz, 2007; Kumazawa et al., 2014; Li et al., 2015). This finding leads us to question whether mitochondrial genome rearrangements show different functional roles and phenotypes and the possibility of other variations occurring in gecko lizard mitogenomes. Information is urgently required for a more comprehensive understanding of these issues. *Cyrtodactylus* comprises a large and diverse genus of gecko lizards in the family Gekkonidae (gekkonids), with over 200 species in Southern and Southeast Asia, Northern Australia and Oceania (Wood et al., 2012; Nazarov et al., 2014). Molecular phylogenies of *Cyrtodactylus* reveal a pattern of diversification and basal divergence that correlates with the India-Asia collision, and the genus is a good candidate for exploring the faunal impacts of this collision (Grismer, Grismer & Chav, 2010; Siler et al., 2010; Welton et al., 2010; Shea et al., 2011). Surprisingly, although the diversity of this lizard is critical, whole mitogenome sequences and structures of *Cyrtodactylus* remain uncertain. The mitogenome sequencing project is thus important to provide a ‘backbone’ for the genus by offering a major framework contribution regarding comparative analyses of biogeography, morphology, diversity, and genome evolution. Here, five complete mitogenome structures of *Cyrtodactylus* as *Cyrtodactylus peguensis*, *C*. *tigroides*, *C*. *thirakhupti, C*. *auribalteatus*, and *C*. *chanhomeae* were characterized. Comparisons between structural variations of the *Cyrtodactylus* mitogenome and other gecko lizards were also discussed.

## Materials and Methods

### Specimen collection and DNA extraction

Individual specimens of each of the five species (*C*. *peguensis*, *C*. *tigroides*, *C*. *thirakhupti*, *C*. *auribalteatus*, and *C*. *chanhomeae*), taken from Nakhon Ratchasima Zoo during 2012–2014, were stored in 100% ethanol and detailed information is presented in Table 1. Individual species were classified based on their morphology (Boulenger, 1893; Bauer, Sumontha & Pauwels, 2003; Pauwels et al., 2004; Sumontha, Panitvong & Deein, 2010). A piece of muscle clipped from each sample was collected to provide a source of DNA. Whole genomic DNA was extracted following the standard salting-out protocol as described previously (Supikamolseni et al., 2015). Animal care and all experimental procedures were approved by the Animal Experiment Committee, Kasetsart University, Thailand (approval no. ACKU-SCI-022), and conducted in accordance with the Regulations on Animal Experiments at Kasetsart University.

**Table 1:** Species used with accession numbers.

Family	Genus	Species	GenBank accession number	Mitochondrial gene arrangement	Reference
Gekkonidae	*Cyrtodactylus*	*Cyrtodactylus peguensis* (CyPeNRZ001)^a^	AP018114	Type I	In this study
Gekkonidae	*Cyrtodactylus*	*Cyrtodactylus thirakhupti* (CyThNRZ001)^a^	AP018115	Type I	In this study
Gekkonidae	*Cyrtodactylus*	*Cyrtodactylus auribaltealus* (CyAuNRZ001)^a^	AP018116	Type I	In this study
Gekkonidae	*Cyrtodactylus*	*Cyrtodactylus chanhomeae* (CyChNRZ001)^a^	AP018117	Type I	In this study
Gekkonidae	*Cyrtodactylus*	*Cyrtodactylus tigroides* (CyTiNRZ001)^a^	AP018118	Type I	In this study
Gekkonidae	*Gekko*	*Gekko chinensis*	NC_027191.1	Type I	Hao, Ping & Zhang (2016)
Gekkonidae	*Gekko*	*Gekko gecko*	NC_007627.1	Type I	Zhou et al. (2006)
Gekkonidae	*Gekko*	*Gekko swinhonis*	NC_018050.1	Type I	Li et al. (2013)
Gekkonidae	*Gekko*	*Gekko japonicus*	NC_028035.1	Type I	Kim et al. (2015)
Gekkonidae	*Gekko*	*Gekko vittatus*	NC_008772.1	Type I	Kumazawa (2007)
Gekkonidae	*Hemidactylus*	*Hemidactylus bowringii*	NC_025938.1	Type I	–
Gekkonidae	*Hemidactylus*	*Hemidactylus frenatus*	NC_012902.2	Type I	Yan, Li & Zhou (2008)
Gekkonidae	*Heteronotia*	*Heteronotia binoei*	NC_010292.1	Type VI	Fujita, Boore & Moritz (2007)
Gekkonidae	*Cnemaspis*	*Cnemaspis limi*	NC_020039.1	Type I	Yan et al. (2014b)
Gekkonidae	*Paroedura*	*Paroedura picta*	NC_028326.1	Type I	Starostová & Musilová (2016)
Gekkonidae	*Tropiocolotes*	*Tropiocolotes tripolitanus*	NC_025780.1	Type II	Kumazawa et al. (2014)
Gekkonidae	*Stenodactylus*	*Stenodactylus petrii*	NC_025784.1	Type III	Kumazawa et al. (2014)
Gekkonidae	*Uroplatus*	*Uroplatus ebenaui*	NC_025783.1	Type IV	Kumazawa et al. (2014)
Gekkonidae	*Uroplatus*	*Uroplatus fimbriatus*	NC_025779.1	Type V	Kumazawa et al. (2014)
Gekkonidae	*Lepidodactylus*	*Lepidodactylus lugubris*	NC_025782.1	Type I	Kumazawa et al. (2014)
Gekkonidae	*Phelsuma*	*Phelsuma guimbeaui*	AB661664.1	Type I	Kumazawa et al. (2014)
Eublepharidae	*Eublepharis*	*Eublepharis macularius*	NC_033383.1	Type I	–
Eublepharidae	*Hemitheconyx*	*Hemitheconyx caudicinctus*	NC_018368.1	Type I	Jonniaux, Hashiguchi & Kumazawa (2012)
Eublepharidae	*Goniurosaurus*	*Goniurosaurus luii*	NC_026105.1	Type I	Li et al. (2015)
Eublepharidae	*Coleonyx*	*Coleonyx variegatus*	NC_008774.1	Type I	Kumazawa (2007)
Phyllodactylidae	*Phyllodactylus*	*Phyllodactylus unctus*	NC_020038.1	Type I	Yan et al. (2014a)
Phyllodactylidae	*Tarentola*	*Tarentola mauritanica*	NC_012366.1	Type I	Albert et al. (2009)
Pygopodidae	*Aprasia*	*Aprasia parapulchella*	NC_024557.1	Type I	MacDonald et al. (2005)
Sphaerodactylidae	*Teratoscincus*	*Teratoscincus keyserlingii*	AY753545.1	Type I	Macey et al. (2005)
Sphaerodactylidae	*Teratoscincus*	*Teratoscincus roborowskii*	KP115216.1	Type I	–
Iguanidae	*Iguana*	*Iguana iguana*	NC_002793.1	–	Janke et al. (2001)
Scincidae	*Plestiodon*	*Plestiodon egregius*	NC_000888.1	–	Kumazawa & Nishida (1999)
Varanidae	*Varanus*	*Varanus salvator*	NC_010974.1	–	Castoe et al. (2008)

**Notes.**

a Samples from Nakhon Ratchasima Zoo, Thailand.

### Complete mitogenome sequencing

Complete mitogenome sequences were obtained using a PCR (polymerase chain reaction)-based strategy to amplify overlapping mitochondrial fragments. Forty-six PCR primer pairs were designed based on five squamate reptile mitogenome sequences: *Gekko chinensis* (NC_027191.1), *G. gecko* (NC_007627.1), *G. japonicas* (NC_028035.1), *G. swinhonis* (NC_018050.1), and *Hemidactylus bowringii* (NC_025938.1). Nineteen primer pairs were also taken from Kumazawa & Endo (2004) (Table S1). PCR amplification was performed using 20 µl of 1×ThermoPol buffer containing 1.5 mM MgCl_2_, 0.2 mM dNTPs, 5.0 µM of primers, 0.5 U of *Taq* polymerase (Apsalagen Co. Ltd., Bangkok, Thailand), and 25 ng of genomic DNA. PCR conditions were as follows: initial denaturation at 94 °C for 3 min, followed by 35 cycles of 94 °C for 30 s, 45–60 °C for 30 s, 72 °C for 1 min 30 s, and then a final extension at 72 °C for 5 min. PCR products were detected by electrophoresis on 1% agarose gels. No multiple bands were found in any of the PCR products. PCR products were purified using FavorPrep GEL/PCR Purification Mini Kit (Favorgen Biotech Corp., Ping-Tung, Taiwan), and nucleotide sequences of the DNA fragments were determined using a 3500 Genetic Analyzer (Life Technology, California, USA). BLASTn and BLASTx programs (http://blast.ncbi.nlm.nih.gov/Blast.cgi) were used to search for nucleotide sequences in the National Center for Biotechnology Information database to confirm the identity of the DNA fragments amplified in the present study.

### Nucleotide sequence annotation and analysis

Sequence assembly was performed to combine all overlapping PCR fragments into one contig strand using SeqScape software v 2.5 (Life Technology, California, USA) and re-checked carefully by visual inspection to ensure accuracy of the variable sites and avoid the problems of repeated sequences identified by the program in the CR. Gene structures of all mitogenomes were annotated using web-based MITOS (Bernt et al., 2013) with manual inspections to compare DNA or amino acid sequences with known sequences from several gecko lizards. ExPASy-translate tool (Gasteiger et al., 2003) was used to characterize the nucleotide sequences of encoded genes. To identify mitochondrial tRNA genes, the nucleotide sequences were searched for regions which could form characteristic secondary structures using tRNA Scan-SE 2.0 by parameter sequence source: vertebrate mitochondrial genome search mode (http://lowelab.ucsc.edu/tRNAscan-SE), the RNAfold web server (http://rna.tbi.univie.ac.at/cgi-bin/RNAWebSuite/RNAfold.cgi) and UNFOLD (http://unafold.rna.albany.edu/) under fold algorithms as basic options is minimum free energy (MFE) and partition function (Pereira et al., 2008). Overlapping regions and intergenic spacers between genes were counted manually. The CR domains comprising the extended termination associated sequence (ETAS) domain, central conserved region (CCR) domain, and conserved sequence block (CSB) domain were characterized following Douzery & Randi (1997). Tandem repeated sequences in CR were identified using the program ‘Tandem Repeats Finder’ with default parameters (Benson, 1999). The most thermodynamically stable putative secondary structures of VNTR were determined using the UNFOLD and RNAfold web servers. All nucleotide sequences were then deposited in the DNA Data Bank of Japan (DDBJ) (Table 1). Base composition and codon usage were analyzed using the default parameters of Molecular Evolutionary Genetics Analysis 7 (MEGA7) software (Center for Evolutionary Functional Genomics, The Biodesign Institute, Tempe, AZ, USA; Kumar, Stecher & Tamura, 2016). The A +T% and G +C% values, and the AT-skew and GC-skew (Perna & Kocher, 1995) for the H-strand were also calculated for five *Cyrtodactylus* mitogenomes, another 25 gecko lizard mitogenomes, and *Iguana iguana* (NC_002793.1), *Plestiodon egregious* (NC_000888.1), and *Varanus salvator* (NC_010974.1) mitogenomes as the outgroup. Values obtained were subsequently mapped as a scatter plot.

### Phylogenetic placement

Multiple sequence alignment of concatenated heavy-strand encoded protein-coding genes was performed on 33 sequences for 30 gecko lizards with the outgroup (*I. iguana*, *P.  egregious*, and *V. salvator*) using the default parameters of MEGA 7 software. Additionally, the *ND6* gene was used separately to perform alignment from phylogenetic analyses of the datasets as it was encoded by the L-strand and possessed base composition bias (Dong & Kumazawa, 2005; Wang et al., 2009). The dataset of sequence lengths of concatenated heavy-strand encoded protein-coding genes ranged from 10,766 to 10,899 bp, while *ND6* sequence lengths ranged from 510 to 546 bp. All unalignable and gap-containing sites were carefully removed and trimmed from the datasets. Datasets of aligned concatenated heavy-strand encoded protein-coding genes and *ND6* fragments showed 10,813 and 548 nucleotides, respectively. Level of sequence divergence between species was estimated using uncorrected pairwise distances (*p*-distances) as implemented in MEGA7. We also performed multiple sequence alignment comprising gap-containing sites (as insertion and deletion) using the GUIDANCE2 Server (Sela et al., 2015). Datasets of aligned concatenated heavy-strand encoded protein-coding genes and *ND6* fragments showed 11,322 and 607 nucleotides, respectively. Phylogenetic analysis was performed using Bayesian inference (BI) and maximum likelihood (ML). The best-fit model of DNA substitution was determined for each gene using Kakusan4 (Tanabe, 2011). The GTR+I+G model gave the best-fit of each locus for BI and ML analyses. BI analysis was performed with MrBayes v 3.2.6 (Huelsenbeck & Ronquist, 2001). The Markov chain Monte Carlo process was used to run four chains simultaneously for one million generations. Log likelihood and parameter values were accessed through Tracer version 1.6 (Rambaut et al., 2015). After the log-likelihood value stabilized, a sampling procedure was performed every 100 generations to obtain 10,000 trees and a majority-rule consensus tree with average branch lengths was generated. All sample points prior to attaining convergence were discarded as burn-in, and the Bayesian posterior probability in the sampled tree population was calculated as a percentage. ML analyses for all datasets were performed using RAxML (Stamatakis, 2014), followed by executing 10 runs of random additional sequences generating 1,000 rapid bootstrap replicates. By combining the definite phylogenetic relationships of different species with the distribution pattern of mitogenome types in gecko lizards, evolutionary processes of the mitogenome structure were determined. Mitochondrial gene rearrangements with similar components and order were classified as identical. All mitogenome types were visualized by linearized organization and drawn by OrganellarGenomeDRAW v 1.2 (OGDraw) (Lohse et al., 2013).

## Results

**Figure 1:**
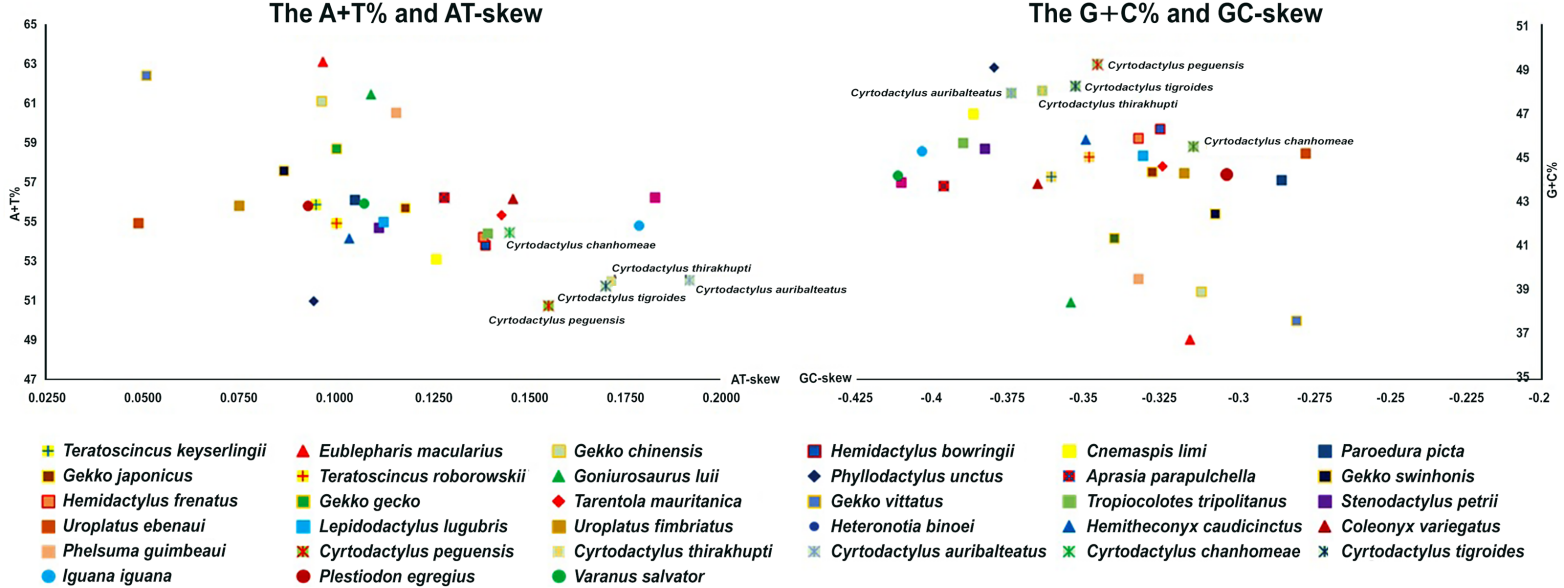
AT-skew versus A+T% and GC-skew versus G+C% in *Cyrtodactylus* and gecko lizard mitochondrial genomes (mitogenomes). Values are calculated on heavy strands for the full length of mitogenomes. The *x*-axis indicates the skew values, the *y*-axis provides the A+T% or G+C% values.

Total mitogenome sequences of the five *Cyrtodactylus* species ranged between 16,795 and 17,068 bp in length. Their genome content comprised 13 protein-coding genes, two rRNA genes, 22 tRNA genes, and the CR (Table 2). There were 28 genes encoded in the majority strand (H-strand) and nine in the minority strand (L-strand), whereas the CR was surrounded by tRNA-Pro and tRNA-Phe in the genome. Average A+T% of the mitogenomes was 52.2 ± 0.61. Highest A+T% was recorded by *C*. *chanhomeae* (54.5%) with the lowest from *C*. *peguensis* (50.8%). By contrast, the average AT-skew was 0.17 ± 0.01, ranging from 0.15 (*C*. *chanhomeae*) to 0.19 (*C*. *auribalteatus*), with the average GC-skew −0.35 ± 0.01, ranging from −0.37 (*C*. *auribalteatus*) to −0.31 (*C*. *chanhomeae*) (Fig. 1).

**Table 2:** Summary of mitochrondrial genome composition of five *Cyrtodactylus* species.

Species	GenBank accession number	Size (bp)	CR^a^ size (bp)	%Nucleotide composition				G+C% content	GC-skew	A+T% content	AT- skew
				A	T	G	C				
*Cyrtodactylus peguensis*	AP018114	16,988	1,653	29.4	21.5	16.1	33.1	49.2	−0.3455	50.8	0.1552
*Cyrtodactylus thirakhupti*	AP018115	16,795	1,454	30.4	21.5	15.3	32.8	48.0	−0.3638	52.0	0.1715
*Cyrtodactylus auribalteatus*	AP018116	16,795	1,445	31.1	21.1	15.0	32.9	47.9	−0.3737	52.1	0.1916
*Cyrtodactylus chanhomeae*	AP018117	17,068	1,728	31.2	23.3	15.6	29.9	45.5	−0.3143	54.5	0.1450
*Cyrtodactylus tigroides*	AP018118	16,929	1,557	30.3	21.5	15.6	32.6	48.2	−0.3527	51.8	0.1699

**Notes.**

a CR indicates control region.

Boundaries between the protein-coding genes were determined by aligning their sequences and identifying the transcription initiation and termination codons with those of known gecko lizards. Common start codons of protein-coding genes were ATG, ATA, and GTG (Table S2). Nine protein-coding genes ended with complete stop codons: TAA, TAG, AGA, and AGG, except for *ND3* in all *Cyrtodactylus* species and *Cytb* in *C*. *auribalteatus* with TA, whereas the remaining protein-coding genes (*COII*, *COIII*, and *ND4*) ended with the incomplete stop codon T, which appeared to be commonly created by post-transcriptional polyadenylation in vertebrate mitogenomes (Ojala, Montoya & Attardi, 1981). The longest protein-coding gene in all *Cyrtodactylus* species was *ND5*, whereas the shortest was *ATPase8.* Average comparison of nucleotide diversity among the five *Cyrtodactylus* mitogenomes was determined at 20.6 ± 0.3%. Highest level of sequence diversity (*p*-distance) in protein-coding genes was recorded for *ATPase8* at 40.9% (between *C*. *peguensis* and *C*. *chanhomeae*), while the lowest was *COIII* at 14.7% (between *C. tigroides* and *C. auribaltealus*). Nucleotide diversity of *ND2* was 21.0 ± 1.2%, *COI* 18.3 ± 0.9%, and *Cytb* 20.2 ± 1.1%. These three genes are often used for molecular phylogenic analysis (Castoe et al., 2008; Supikamolseni et al., 2015; Laopichienpong et al., 2016). Comparing nucleotide sequences of the five *Cyrtodactylus* mitogenomes with those of other gecko lizards provided a clear pattern of nucleotide diversity for whole mitogenomes, *ND2*, *COI*, and *Cytb* in the families Gekkonidae, Eublepharidae, Pygopodidae, Phyllodactylidae and Sphaerodactylidae, and infraorder Gekkota (Table 3).

**Table 3:** Comparison of sequence divergence among gecko lizards based on whole mitochondrial genome and three mitochondrial gene sequences (*ND2*, *COI*, and *Cytb*).

Sample group	Average genetic distance ±standard error			
	Mitochondrial genome	*ND2*	*COI*	*Cytb*
*Cyrtodactylus* (5)^a^	0.206 ±0.003	0.210 ±0.012	0.183 ±0.009	0.202 ±0.011
Intrafamily Gekkonidae (16)^a^	0.334 ±0.004	0.346 ±0.014	0.243 ±0.010	0.298 ±0.013
Intrafamily Eublepharidae (4)^a^	0.306 ±0.003	0.350 ±0.014	0.231 ±0.010	0.269 ±0.012
Intrafamily Phyllodactylidae(2)^a^	0.313	0.362	0.236	0.299
Intrafamily Pygopodidae (1)^a^	–	–	–	–
Intrafamily Sphaerodactylidae (2)^a^	0.200	0.171	0.215	0.182
Infraorder Gekkota (30)^a^	0.315 ±0.005	0.351 ±0.015	0.239 ±0.010	0.274 ±0.015

**Notes.**

a Integers in parenthesis indicate the number of species analyzed.

**Table 4:** Summary of a non-coding control region composition of five *Cyrtodactylus* species.

Species	GenBank accession number	CR^a^		ETAS^b^		CCR^c^			CSB^d^				
		CR^a^ size (bp)	A+T% content	ETAS^b^ size (bp)	A+T% content	CCR^c^ size (bp)	A+T% content	CSB-F size (bp)	CSB^d^ size (bp)	A+T% content	CSB-1 size (bp)	CSB-2 size (bp)	CSB-3 size (bp)
*Cyrtodactylus peguensis*	AP018114	1,653	57.7	496	76.6	312	52.9	20	845	48.4	21	18	18
*Cyrtodactylus thirakhupti*	AP018115	1,454	59.4	495	76.8	311	51.5	20	648	49.9	21	18	18
*Cyrtodactylus auribalteatus*	AP018116	1,445	57.7	566	70.9	246	48.0	20	633	49.8	21	18	18
*Cyrtodactylus chanhomeae*	AP018117	1,728	62.5	647	74.8	254	56.7	20	827	54.7	21	18	18
*Cyrtodactylus tigroides*	AP018118	1,557	57.2	492	73.8	314	54.2	20	751	47.8	21	18	18

**Notes.**

a CR indicates control region.

b ETAS indicates extended termination associated sequence domain.

c CCR indicates central conserved region domain.

d CSB indicates conserved sequence block domain.

A total of 22 tRNAs were interspersed throughout the mitogenome. Their size ranged from 54 bp (tRNA-Tyr) to 74 bp (tRNA-Phe). Most tRNAs could be folded into the canonical cloverleaf secondary structure, except for tRNA-Cys and two tRNA-Ser which appeared to lack the dihydrouridine arm. A characteristic stem and loop structure of an origin for light-strand replication (O_L_) was present between the tRNA-Asn and tRNA-Cys of the WANCY tRNA gene cluster. The size of 16S rRNA ranged from 1,533 bp (*C*. *chanhomeae*) to 1,545 bp (*C*. *tigroides*), with 12S rRNA ranging from 946 (*C*. *peguensis*) to 958 (*C*. *thirakhupti*). Total numbers of intergenic spacers were 13, 12, 10, 9, and 7 in the mitogenomes of *C*. *tigroides, C*. *chanhomeae*, *C. peguensis, C*. *auribalteatus*, and *C*. *thirakhupti*, respectively (Tables S3–S7). Interestingly, the 28 bp spacer between *COI* and tRNA-Ser was observed in *C. tigroides* but not for other *Cyrtodactylus* species.

The size of the CR for the five *Cyrtodactylus* species ranged from 1,445 to 1,728 bp (Table 4), and average A+T% was 58.9 ± 1.0. Lengths of the ETAS domain varied from 492 bp to 647 bp among the five *Cyrtodactylus* species. The TAS (termination-associated sequence) was identified by the conserved pentanucleotides (5′-TACAT-3′). The TAS elements were also located in repeated sequences of VNTR. These tandem repeats were identified in the ETAS domain containing six repeated sequences of a 75-bp named 75-bp box in *C*. *peguensis*, *C*. *tigroides* and *C*. *thirakhupti,* and eight 75-bp boxes in *C*. *chanhomeae*. However, two repeated sequences of a 225-bp box were found in *C*. *auribalteatus*. The tandem repeats were also able to form an “inverted repetitions” type structural conformation which was very similar to hairpins (Fig. S1). Secondary structures of single repeated sequences were retrieved based on the minimum free energy model (Zuker & Stiegler, 1981). The CCR domain ranged from 246 to 314 bp in length in all *Cyrtodactylus*. The consensus sequences of CSB-F were recognized as “CHCGRGAAACCAKCRACCCS”. Moreover, the size of the CSB domain ranged from 633 bp (*C*. *auribaltealus*) to 845 bp (*C*. *peguensis*). This domain contained three conserved blocks: CSB-1 (5′-KTTMATGCTCGAWRGACATAY-3′), CSB-2 (5′-AAACCCCCCTTACCCCCC-3′), and CSB-3 (5′-CGCCAAACCCCTAAAACG-3′) involving the regulation of replication and transcription (Clayton, 1991; Shadel & Clayton, 1997). Sequence similarities of CSB-2 and CSB-3 among *Cyrtodactylus* were higher than those of CSB-1. Microsatellite motifs: (TA)_n_ and/or (CA)_n_ were found between CSB-1 and CSB-2, or between CSB-3 and tRNA-Phe in *Cyrtodactylus* (Table S8).

**Figure 2:**
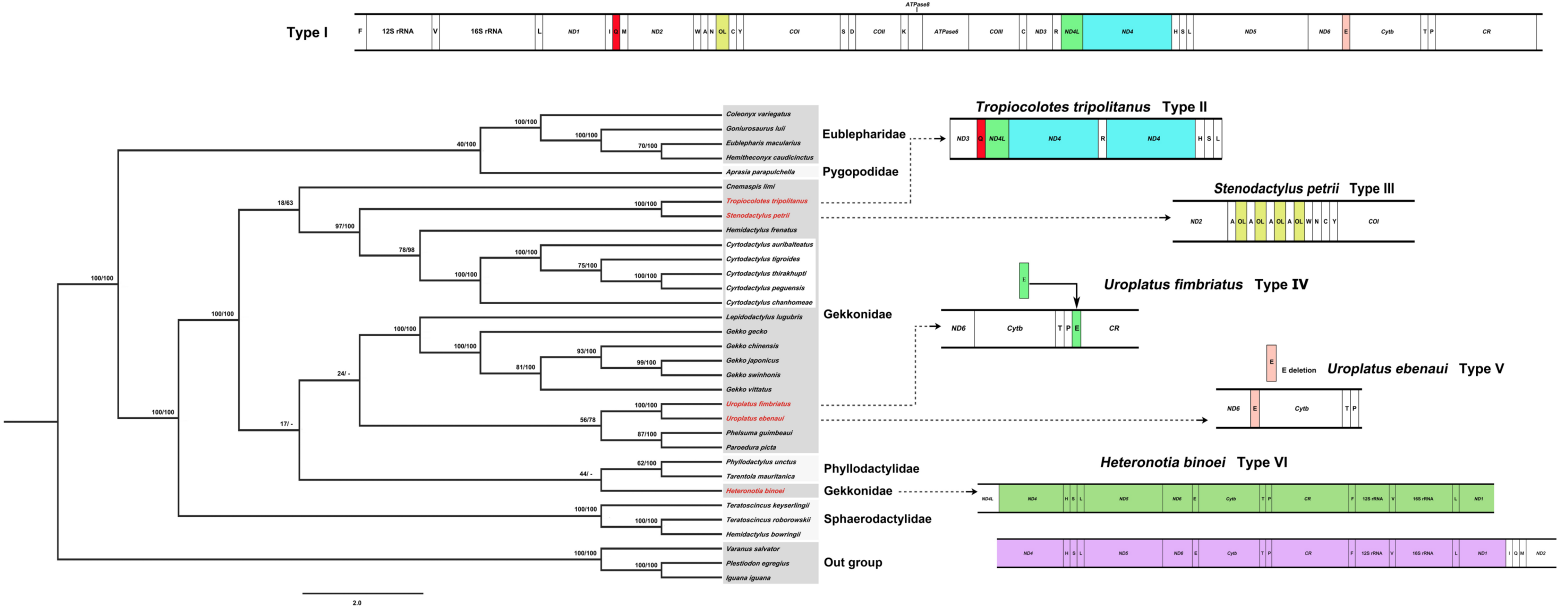
Phylogenetic relationship among 30 gecko lizards with *Iguana iguana*, *Plestiodon egregious* and *Varanus salvator* as the outgroup, constructed from Bayesian inference analysis using concatenated heavy-strand encoded protein-coding gene sequences. Support values at each node are bootstrap values from maximum likelihood (ML) (left) and Bayesian posterior probability (right). Asterisk (*) indicates full support (100%, 1.0) in both analyses, and hyphen (-) indicates no support. Detailed information of all gecko lizards is presented in Table 1. Mitochondrial gene arrangement types for gecko lizards are shown on the right hand side.

Phylogenetic positions of the five *Cyrtodactylus* species in gecko lizards were analyzed based on the concatenated heavy-strand encoded protein-coding genes and the *ND6* gene with/without gap-containing site datasets from 30 gecko lizards. Both datasets showed highly similar tree topology (Fig. 2, Fig. S2). The pattern of phylogenies of the mitogenome sequence was monophyletic, with most species clustered in each taxonomic family. Five *Cyrtodactylus* species were identified within the same group. These results showed similar tree topology and strongly supported the established gecko lizard relationship (Pyron, Burbrink & Wiens, 2013; Kim et al., 2015).

## Discussion

Using the PCR method, complete mitogenomes of the five *Cyrtodactylus* species were determined for genome size as 16–17 kb, similar to those of other gecko lizards (Zhou et al., 2006; Kumazawa et al., 2014; Kim et al., 2015). The GC-skew values were negative in all gecko lizard mitogenomes and this phenomenon is highly conserved among vertebrates (Zhou et al., 2006; Kumazawa et al., 2014; Hao, Ping & Zhang, 2016). The GC-skew values were subjected to statistical evaluation of the asymmetric distribution of the two complementary base pair DNA strands, which suggest a higher content of C compared with G nucleotides. Comparison of A+T% for gecko lizard mitogenomes showed that the *Cyrtodactylus* mitogenome exhibited a low value (52.2 ± 0.6), whereas values of the AT-skew of *Cyrtodactylus* were higher than other gecko lizards, indicating a higher occurrence of A compared to T nucleotides. A large base composition bias arose in the *Cyrtodactylus* lineage after it diverged from a common ancestor around 65 million years ago in the late Paleogene (Wood et al., 2012). Although sequence diversity (*p*-distances) of *ATPase8* was high (>40%) among *Cyrtodactylus* species in this study, interspecific sequence divergences of *ND2, COI,* and *Cytb* as standard DNA barcodes for squamate reptiles (Castoe et al., 2008; Supikamolseni et al., 2015; Laopichienpong et al., 2016) were about 20% (Table 3). Gene coding for the subunits of cytochrome oxidase and cytochrome *b* were conserved, while the most variable genes were the *ND* and *ATPase* (Meyer, 1993). Interspecific sequence divergences in *Cytb* were about 19–27% in gecko lizards (Lamb, Bauer & McEachran, 2001), while sequence divergences of congeneric variation were between 4.3% and 28.7% (average = 21.0 ± 4.2%) for the *COI* gene (Nguyen et al., 2014) and 21% for the *ND2* gene in *Cyrtodactylus* and *Geckoella* (Shea et al., 2011; Agarwal et al., 2014; Agarwal & Karanth, 2015). This result agreed with sequence divergence of the three genes among *Cyrtodactylus* species and gecko lizards in this study, suggesting that the three genes are effective for identifying species in *Cyrtodactylus* and gecko lizards.

### Mitochondrial gene rearrangement of *Cyrtodactylus* species in gecko lizards

Generally, organization of the 37 genes and the CR of most gecko lizards tend to be conserved in squamate reptiles (Zhou et al., 2006; Kumazawa et al., 2014; Kim et al., 2015; Hao, Ping & Zhang, 2016). However, various rearrangement patterns of mitogenomes have been found in snakes (Yan, Li & Zhou, 2008), varanid lizards (Amer & Kumazawa, 2008), and acrodont lizards (Okajima & Kumazawa, 2010) as a consequence of the shuffling of tRNA gene clusters, translocations and/or duplications of genes, and gene loss (Anderson et al., 1981; Fujita, Boore & Moritz, 2007; Kumazawa et al., 2014; Li et al., 2015). Comparison of gecko lizard mitogenomes revealed six types of mitochondrial gene rearrangements as I, II, III, IV, V, and VI based on available gecko lizard mitogenome sequences. Type I is distributed in most gecko lizards (80%) including *Cyrtodactylus* species. Types II and III are found in *Tropiocolotes tripolitanus* and *Stenodactylus petrii*, respectively. Type II was derived from the Type I by duplication of tRNA-Gln and *ND4*, whereas Type III showed multiple copies of O_L_ within the WANCY cluster. Type IV was observed in *Uroplatus fimbriatus*, resulting from the translocation of tRNA-Glu from 5′ proximal *ND 6* to 5′ proximal tRNA-Pro (Fig. 2). Deletion of tRNA-Glu was found in *Uroplatus ebenaui* as Type V, while duplication of 5′ proximal *ND4*- to 5′ proximal tRNA-Ile was observed in *Heteronotia binoei* as Type VI. Our phylogenetic placement indicated that gecko lizard mitogenome Types II–VI were found at the terminus of the tree in Gekkonidae, suggesting that structural variation of mitogenomes has occurred independently in gekkonids. Rare changes of mitochondrial gene rearrangement in gecko lizard lineages may indicate the possibility of homoplasy-free datasets. However, many family-level taxa are represented by a few species; thus, more gecko lizard mitogenome sequences are required to better understand the evolutionary history in this lineage.

### Dynamics of CR in *Cyrtodactylus*

Although the CRs of the five *Cyrtodactylus* species were mainly composed of high A+T%, distribution of AT was not homogenous among the three domains. The AT content of the CSB domain was lower than the ETAS and CCR domains (Table 4). The size of CRs varied from 1,445 to 1,728 bp among *Cyrtodactylus* species and tallied with those of gecko lizards. This feature was mainly caused by the duplication of tandem repeat at VNTR and frequently occurs in ETAS and/or CSB domains (Larizza et al., 2002). In the ETAS domain, different copy numbers of the 75-bp box tandem repeat were found in most *Cyrtodactylus* with at least 76% similarities, except for *C*. *auribalteatus* containing 225-bp box. These results collectively suggest that the 75-bp box tandem repeat was present in ancestral mitogenomes before divergence of *Cyrtodactylus* species. Here, the 75-bp box is designated as 75-bp box family. Variable copy numbers have resulted from independent duplication in each species, a factor commonly observed in other vertebrate groups (Zhou et al., 2006; Kumazawa et al., 2014; Starostová & Musilová, 2016).

**Figure 3:**
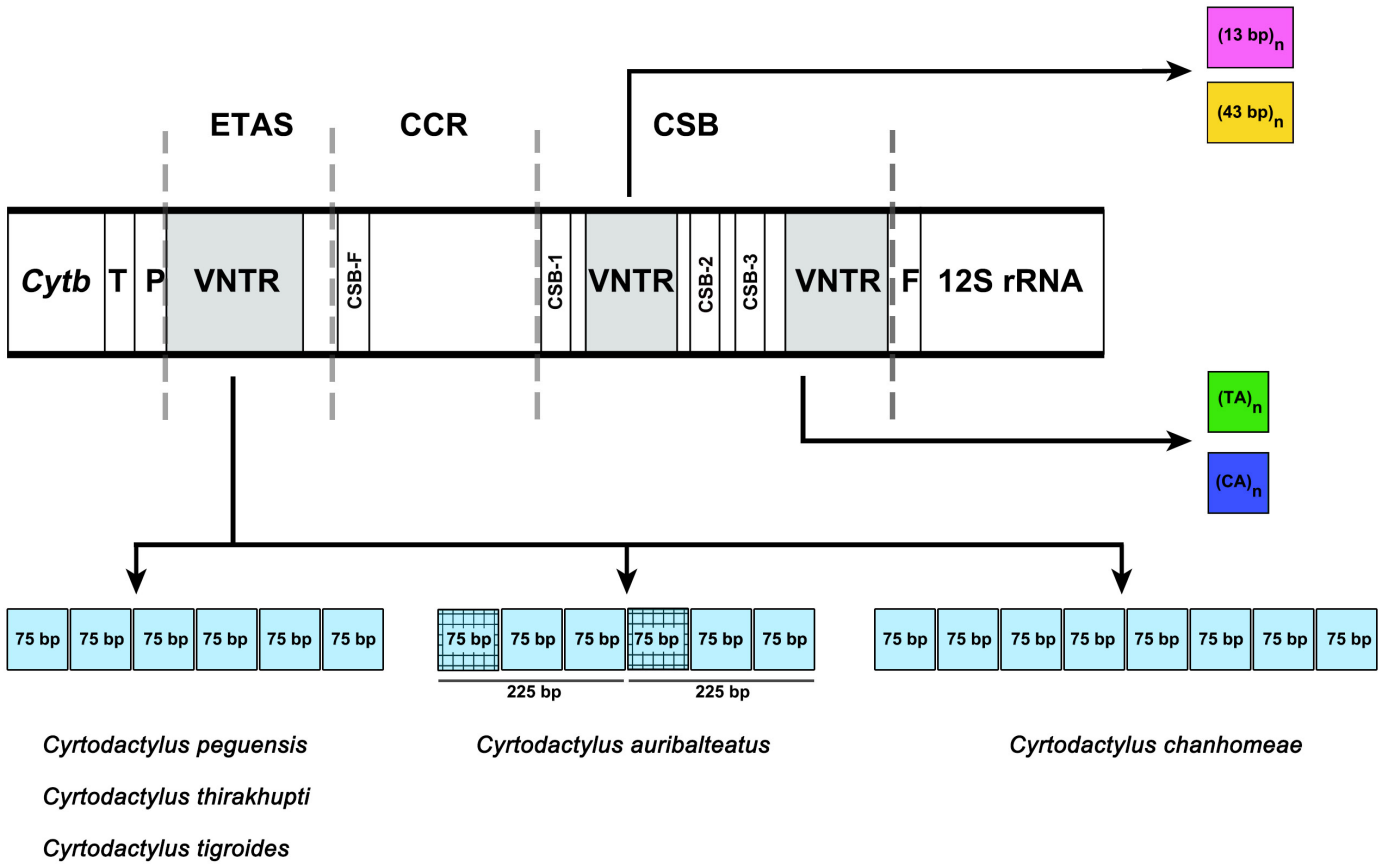
Schematic representation of a non-coding control region (CR) structure of *Cyrtodactylus* species. Abbreviations: TAS, Termination Associated Sequence; CSB, Conserved Sequence Block; VNTR, variable number of tandem repeats. The square color box indicates a repeated sequence. Same color boxes show grid pattern box indicating sequence similarity of more than 65%.

Comparison of 75-bp and 225-bp boxes among *Cyrtodactylus* species revealed 69% similarities. The 225-bp box is likely to contain two 75-bp boxes tandemly and one 75-bp box-like (Fig. 3). This result suggests that the presence of 225-bp box resulted from duplication of 75-bp boxes with nucleotide substitution, followed by homogenization and fixation in *C*. *auribalteatus* after it diverged from *Cyrtodactylus*. Interestingly, both the sequence of 75-bp and 225-bp boxes could form stable secondary structures that might be responsible for increasing possible misalignment (H-strand and L-strand), involving rearrangement in CR during DNA replication of mitochondrial DNA (Broughton & Dowling, 1997; Pereira et al., 2008). Secondary structures of VNTR are believed to be termination sequences in the replication process in fish (Terencio et al., 2013). Although the function of these secondary structures remains completely unknown, they were probably retained in *Cyrtodactylus* under selective pressure. Structural and functional studies are required to explain this molecular mechanism. To investigate the distribution of the 75-bp box family in gecko lizards, a comparative search (BLAST) was conducted for similar sequences housed in the GenBank. A tandem repeat with 55–75% similarities was found in most gecko lizards, even for Eublepharidae and Pygopodidae as the basal group. This result suggests that the tandem repeat of 75-bp box family was acquired in the genome of the common ancestor of gecko lizards and evolved gradually through independent nucleotide mutation in each lineage.

The CCR domain is highly conserved among gecko lizards compared to ETAS and CSB domains. The CSB-F block characterized the CCR domain in *Cyrtodactylus* and different levels of similarity (59.26–96.15%) were observed when compared with other gecko lizards. However, two blocks (CSB-E and/or CSB-D) observed in other vertebrates were not identified in *Cyrtodactylus* and other gecko lizards (Anderson et al., 1981; Broughton & Dowling, 1994; Lee et al., 1995). This result suggests plausible plasticity across vertebrates concerning the CSB-F, CSB-E, and CSB-D blocks. The CSB domain in *Cyrtodactylus* and gecko lizards is characterized by the three conserved blocks CSB-1, CSB-2, and CSB-3 which involve regulation of replication and transcription (Li et al., 2013; Kumazawa et al., 2014). These three blocks have also been identified in vertebrate mitogenomes (Liu, 2002; Brehm et al., 2003; Zhang et al., 2009; Xiong et al., 2010). The sequence of CSB-3 blocks is the most highly conserved in *Cyrtodactylus* and other gecko lizards, whereas the CSB-1 block showed the most variability. Repeated sequences as minisatellites and/or microsatellites (TA)_n_ or (CA)_n_ were found between CSB-1 and CSB-2 or between CSB-3 and tRNA-Phe in *Cyrtodactylus* and other gecko lizards (Table S8). Minisatellites were found in four gecko lizards (*Hemitheconyx caudicinctus*, *Gekko japonicas*, *U*. *fimbriatus*, and *U*. *ebenaui*) which did not contain tandem repeats in the ETAS domain. This result suggests that repeated sequences are detected in at least ETAS or CSB domains of CR. Thus, VNTRs can be used as molecular markers to provide phylogenetic information in population genetics, species identification, genetic diversity, and conservation (Zardoya & Meyer, 1998; Zhang et al., 2009). However, VNTRs differ in length, copy number, and base composition even in very closely related species, and may be shared by distantly related species but not by close ones, such as in *C*. *auribalteatus*. This result suggests that the presence/absence of repeated sequences might not be a reliable phylogenetic marker.

## Conclusions

Here, complete mitogenomes of five *Cyrtodactylus* species were sequenced and characterized with those of gecko lizards to examine their structural patterns. Six types of mitochondrial gene rearrangements indicated low diversification in gecko lizards, whereas nucleotide sequence diversity among gecko lizards was exhibited at considerably high levels and might be useful as a tool to resolve taxonomic controversies. The CR evolutionary pattern strongly suggests high conservation level of the 75-bp repeated sequence family in gecko lizards. These repeated sequences also form secondary structures, indicating that repeated sequence families played an important role under selective pressure. This might have resulted in involvement of these structures in mitogenome replication which caused rearrangements in CR. However, since many family-level taxa are represented by a limited number of species, the detailed knowledge of the phylogeny of gecko lizard mitogenomes requires further sampling and sequencing exports.

##  Supplemental Information

10.7717/peerj.6121/supp-1 Figure S1 Secondary structures of 75-bp repeated sequence family of five *Cyrtodactylus* species formed using RNAfold web server (http://rna.tbi.univie.ac.at/cgi-bin/RNAWebSuite/RNAfold.cgi)

10.7717/peerj.6121/supp-2 Figure S2 Phylogenetic relationship among 30 gecko lizards with *Iguana iguana*, *Plestiodon egregious* and *Varanus salvator* as the outgroup, constructed from Bayesian inference analysis using *ND6* gene sequences. Support values at each node are bootstrap values from maximum likelihood (ML) (left) and Bayesian posterior probability (right). An asterisk (*) indicates full support (100%, 1.0) in both analyses and a hyphen (-) indicates no support. Detailed information of all gecko lizards is presented in Table 1.

10.7717/peerj.6121/supp-3 Table S1 Primers used for PCR amplification and sequencing of complete mitochrondrial genome of five *Cyrtodactylus* species

10.7717/peerj.6121/supp-4 Table S2 Usage of initial and termination codons in five *Cyrtodactylus* species

10.7717/peerj.6121/supp-5 Table S3 Mitochondrial genome organization and features in *Cyrtodactylus peguensis*

10.7717/peerj.6121/supp-6 Table S4 Mitochondrial genome organization and features in *Cyrtodactylus thirakhupti*

10.7717/peerj.6121/supp-7 Table S5 Mitochondrial genome organization and features in *Cyrtodactylus auribalteatus*

10.7717/peerj.6121/supp-8 Table S6 Mitochondrial genome organization and features in *Cyrtodactylus chanhomeae*

10.7717/peerj.6121/supp-9 Table S7 Mitochondrial genome organization and features in *Cyrtodactylus tigroides*

10.7717/peerj.6121/supp-10 Table S8 Variability and similarity between repeated sequences in a non-coding control region among gecko lizards

## Additional Information and Declarations

### DNA Deposition

The following information was supplied regarding the deposition of DNA sequences: DDBJ accession numbers: AP018114, AP018115, AP018116, AP018117 and AP018118.

### Data Availability

The following information was supplied regarding data availability: The raw data are provided in the Supplemental Files and shows mitochondrial genome characterization of all species.
