# Supplementary figures and images for: Characterization of five complete Cyrtodactylus mitogenome structures reveals low structural diversity and conservation of repeated sequences in the lineage
Source: PeerJ. 2018 Dec 13;6:e6121. doi: 10.7717/peerj.6121 (PMC6295329; doi:10.7717/peerj.6121)

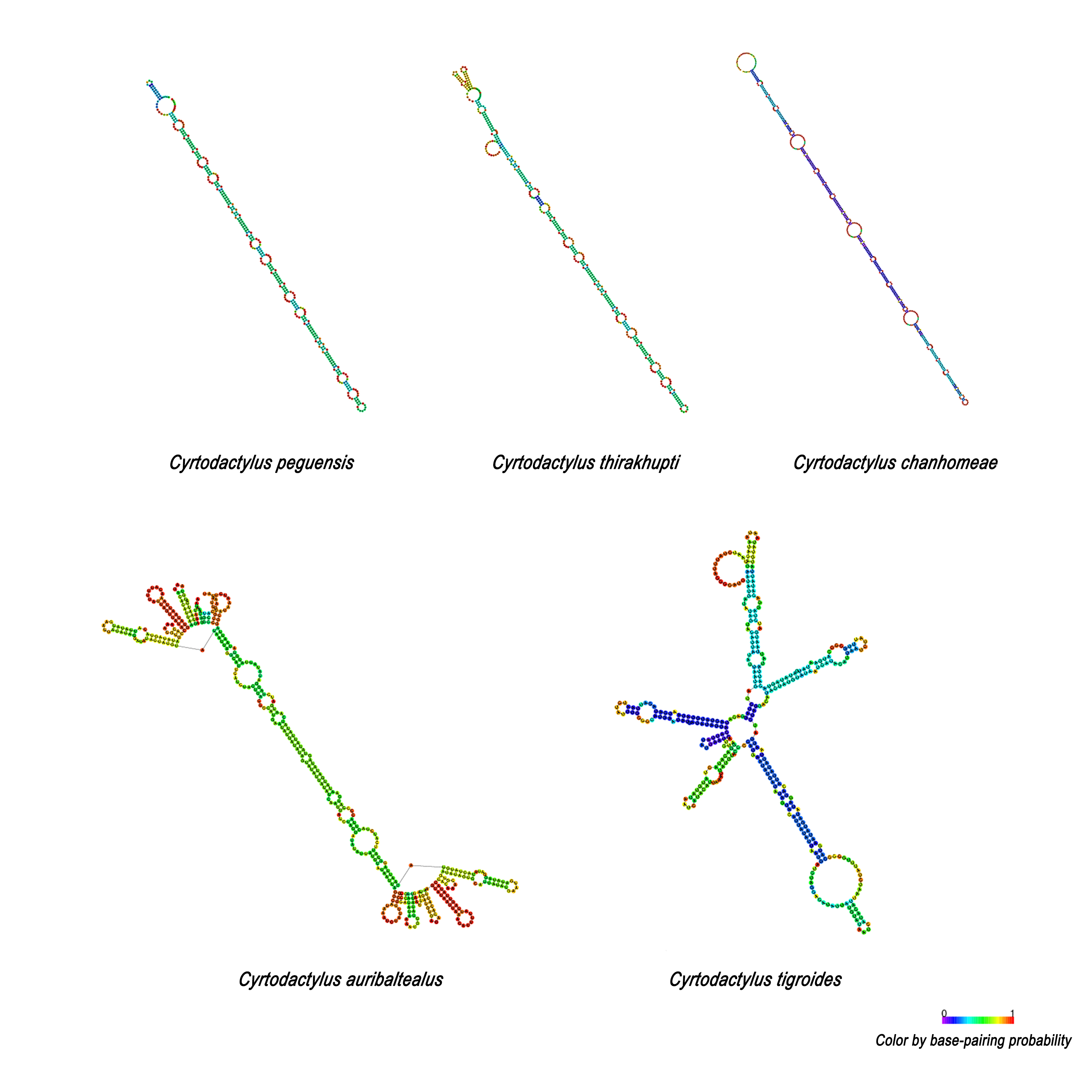

Supplement: Figure S1 [file peerj-06-6121-s001.png]

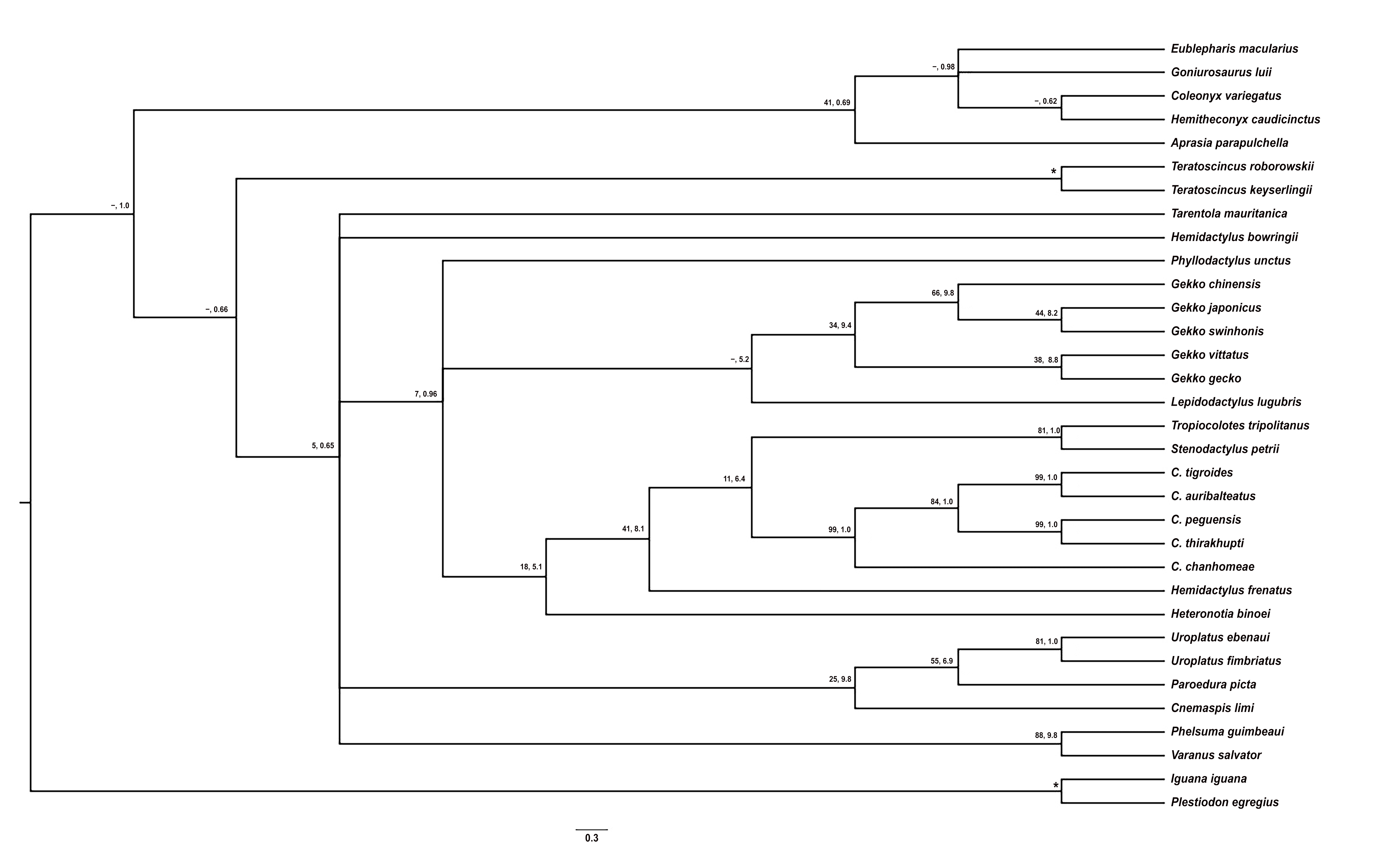

Supplement: Figure S2 — Support values at each node are bootstrap values from maximum likelihood (ML) (left) and Bayesian posterior probability (right). An asterisk (*) indicates full support (100%, 1.0) in both analyses and a hyphen (-) indicates no support. Detailed information of all gecko lizards is presented in Table 1. [file peerj-06-6121-s002.png]
