# Supplementary material for: Characterization of five complete Cyrtodactylus mitogenome structures reveals low structural diversity and conservation of repeated sequences in the lineage
Source: PeerJ. 2018 Dec 13;6:e6121. doi: 10.7717/peerj.6121 (PMC6295329; doi:10.7717/peerj.6121)
Supplement: Table S1 [file peerj-06-6121-s003.docx]

**Table S1** Primers used for PCR amplification and sequencing of complete mitochrondrial genome of five *Cyrtodactylus* species

| No. | Primer name | Primer sequence forward (5'-3') | Primer name | Primer sequence reverse(5'-3') | Reference |
| --- | --- | --- | --- | --- | --- |
| 1 | rCONT-4L | TCGYCAAACCCCWAAAMCGAG | H1858 | TCGATTATAGRACAGGCTCCTCTAG | Kumazawa and Endo 2004 |
| 2 | rl2S-1L | AGGATTAGATACCCTACTA | rl6S-5H | TTTATYRRGYAACCAGCTATC | Kumazawa and Endo 2004 |
| 3 | rl6S-3L | AACCCYYGTACCTYTTGCATCATG | l6sbr-H | CCGGTCTGAACTCAGATCACGT | Kumazawa and Endo 2004 |
| 4 | rl6S-4L | TACTCCAGGGATAACAGCGC | rND1-2H | TCAAATGGKGCTCGRTTDGTTTC | Kumazawa and Endo 2004 |
| 5 | rND1-2L | CAAACMATCTCMTAYGAAGT | rND2-2H | ATTGATGAGWAKGCTATRATTTTTCG | Kumazawa and Endo 2004 |
| 6 | rND2-1L | GCCCCMYTMCACTTCTGA | rAsn-lH | TGGGYGKTTAGCTGTTAAYTA | Kumazawa and Endo 2004 |
| 7 | rTrp-lL | TAAACCARGRGCCTTCAAAG | rCOl-1H | TAGTGGAARTGKGCTACTAC | Kumazawa and Endo 2004 |
| 8 | rCOl-2L | TCWGCCACAATAATYATYGC | uCO2-1H | CCGCAGATTTCTGAGCATTG | Kumazawa and Endo 2004 |
| 9 | uCO2-lL | GGMCAYCAATGATACTGA | uND3-2H | GGGTCRAAKCCRCATTCRTA | Kumazawa and Endo 2004 |
| 10 | uCO3-1L | ATAGTWGACCCMAGCCCATGACC | rND4L-2H | GCTAGGCCAGTRCYTGCTTCRCA | Kumazawa and Endo 2004 |
| 11 | uCO3-3L | GAAGCMGCWGCCTGATACTGACA | rND4-2H | GATGTTAAKCCGTGGGCRATTAT | Kumazawa and Endo 2004 |
| 12 | rND4L-2L | TAACCTTCTCMGCMTGYGAAGC | rCUN-1H | CTTTTACTTGGADTTGCACC | Kumazawa and Endo 2004 |
| 13 | rND4-3L | CCAAAAGCCCAYGTAGARGC | rND5-1H | ACWACTATTGTGCTKGAGTG | Kumazawa and Endo 2004 |
| 14 | rHis-2L | AACAAAAACAYTAGRCTGTG | rND5-2H | ATWGYGTCTTTTGAGTARAAKCC | Kumazawa and Endo 2004 |
| 15 | rND5-1L | TCCAAGCMATYATCTAYAACCG | rND6-4H | ATGTTAGTGGTDTTTGCKTATTC | Kumazawa and Endo 2004 |
| 16 | rND5-3L | YACMYMAACGCCTGAGCCCT | ucytb-1H | GCCCCTCAGAATGATATTTGTCCTCA | Kumazawa and Endo 2004 |
| 17 | rND6-3L | GCAACWGAATAHGCAAATAC | rcytb-1H | GCGTAGGCRAATAGGAAGTATCA | Kumazawa and Endo 2004 |
| 18 | rcytb-2L | TGAGGACAAATATCMTTCTGAGG | rPro-1H | TTAAAATKCTAGTTTTGG | Kumazawa and Endo 2004 |
| 19 | rThr-2L | YAAAGCMTTGRTCTTGTAA | rCONT-4H | CTCGKTTTWGGGGTTTGRCGA | Kumazawa and Endo 2004 |
| 20 | 17F-ATP8 | ACCACAGCTTCATGCCAATC | 17R-ATP6 | AGCAGGCCAAGGTGTATTG | In this study |
| 21 | 19F-ND3 | GTACAAGCGACTTCCACTCACT | 19R-ND4L | GGGTGTAGGTTCGTTTGCGG | In this study |
| 22 | 30L-CytB | AAGCCCTGGTCTTGTAAACC | 30R-CR | GCCTGACGCTAGTAATAAGGTG | In this study |
| 23 | 31F-CR | ACGAGAAACCATCAACCCGC | 31R-12S | CCATAACGGCAACTTCAGGGC | In this study |
| 24 | 1F-ND1 | GGTAAACTCCGTGCCAGCCA | 1R-16S | AACCAGCTATTGCCAGGCTCG | In this study |
| 25 | 2F-16S | CGAACCCAGACGAGCTACC | 2R-16S | GAAACCGACCTGGATTGCTCC | In this study |
| 26 | 3F-16S | TACCCCAGGGATAACAGCG | 3R-ND1 | TACCAACATTGTCGGGGTATGG | In this study |
| 27 | 4F-t-Ile | GGAAGTGTGCCCGAGAMCC | 4R-ND2 | TTAGGGCTGTGTGTGTGGGC | In this study |
| 28 | 5F-ND2 | TAGCAAGCCTCCCAAGCCT | 5R-CO1 | TAAGGGGGTGTGGTATTGTG | In this study |
| 29 | 15S-ND6F | TGCCGCAGGAAATGGCTCAG | 15S-Cybt | CCGTTGGCGTGCAGGTTTCG | In this study |
| 30 | 21S-G1F | GCCATCAACTTCATCACCACTTGC | 21S-G1R | AGGGTTCCTGGGGTTAGGGC | In this study |
| 31 | 21S-G2F | CGCCTAACCGCAAATCTAACC | 21S-G2R | GCGCCTCATCAGTAGATTGA | In this study |
| 32 | 21S-G3F | GGAATCGCAGTCCCACGTCA | 21F-G3R | GCGGGTTGATGGTTTCTCGT | In this study |
| 33 | 15S-ND4-F | AGCCCTCCAATAAACCAC | 15S-ND4-R | TGCGGCGAGGATTATTGAG | In this study |
| 34 | 15S-CR-F | CGCACCACACTTGCCAACATTC | 15S-CR-R | TATACGCGGCACTGCTATCCTG | In this study |
| 35 | 9S-ND3-F | CTACTGATGAGGTGCTTGCC | 9S-ND3-R | TATTGAGGTGGTCGTTGGAG | In this study |
| 36 | 9S-CR-F | GCTGTCCACCAACTCGTGTA | 9S-CR-R | GGTGTACTGCGATTCCAGAG | In this study |
| 37 | 21S-ND5-F | CTCTCAAAGGACAGCAGCAGT | 21S-ND5-R | TAGCAAGCAGTGGGTGTAGTC | In this study |
| 38 | 21S-G5F | CCACTGCCTTAACGACGAACA | 21S-G5R | ACCCGTCGCCATATTTTGGTG | In this study |
| 39 | 21S-G6F | TTCCTCCACGAAACAGGCTC | 21S-G6R | GAGATAGGAGCCAGAGGTCT | In this study |
| 40 | T-ND2-F | CGCTACGACCARCTAATRCACC | T-ND2-R | CAGGTAGGGTGGCCGAGTA | In this study |
| 41 | T-CO1-F | CCTAGWCTGACGGGCYTCGAT | T-CO1-R | GCRTGGTCGTGCAGRTGTA | In this study |
| 42 | T-ATP-F | GCCARTGCTCMGAAATYTGCGG | T-ATP-R | TGCYTTCGCGGAYGATGTCGC | In this study |
| 43 | T-CO3-F | ATGACCCACCAAGCMCACGC | T-CO3-R | GGCRTGTGTKCGGGCAGTT | In this study |
| 44 | T-ND4-F | TAGACTGRGCAGAATATGGCGGC | T-ND4-R | CCTATGTGGCTRACWGAGGAGT | In this study |
| 45 | 19F-ND3-re | AGTGAGTGGAAGTCGCTTGTAC | 19R-ND3-re | CCGCAAACGAACCTACACCC | In this study |
| 46 | 9S-19F-2 | CTCCTCATCTGCCTCCTGCGACA | 9S-19R-2 | CGATGGGGCTCCCAGGCAGA | In this study |
| 47 | 23S-ND2-F | GCACCACTGACACTACTGTA | 23S-Cytb-R | GGTGAGTGTGGCGTTGTCTA | In this study |
| 48 | 23S-CR-F | CTGGTAAAGCCTCAGTAGC | 23S-CR-R | GTGGCACTGCTATTCAGGTA | In this study |
| 49 | 4S-15S-F | TATCGCCAGGGGGGTTTAC | 4S-ND2-R | ACGTTTTACGGGATCGAGG | In this study |
| 50 | 4S-ND2-F2 | CCTCGATCCCGTAAAACGT | 4S-CO3-R | CTTAGTGAGTGGAAGTCGC | In this study |
| 51 | 4S-ND4-F | CCTCAATCCTCTCGCCAACA | 4S-ND5-R | GTAGTCATGGATGGAGGCCAA | In this study |
| 52 | 4S-ND6-F | GCCCTGCTACACCCACAATAT | 4S-CR1-R | CGTACATGGTCTTTTGGTGTGG | In this study |
| 53 | 23S-ND2-F | TGCACCACTGACACTACTGTAC | 23S-CO1-R | AGATAGGCTGAAGTCCGCTGG | In this study |
| 54 | 23S-CO1T-F | TACTACTGCTTGCCTCCGC | 23S-tLys-R | TCCTTGGAGACGACTGTAGC | In this study |
| 55 | 23S-CO3M-F | GCCTCGCAGTATGATTCCA | 23S-ND4L-R | GATGCAGCGTTAGCCCTGT | In this study |
| 56 | 23S- ND4-F | TGGCGGGTATGGCCTAATTC | 23S-Cytb-R | AGAATCGGGTGAGTGTGGC | In this study |
| 57 | 23-ND5-F | ACGACCTGCTATGATACCAG | 23-CytbA-R | CAGGTTTTGGCAAGAGGCT | In this study |
| 58 | 4-ND2T-F | ACCTGTTAGGGTGCGTCTCC | 4-CO1-R | TGGCTAGGATTACTCCGGTGAG | In this study |
| 59 | 4-CO1T-F | CAACCAAGAATGACTACACGGC | 4-ND4L-R | TGTTTGCGATGCAGCGTTAG | In this study |
| 60 | 4-CO1-F | CACCTGTTAGGGTGCGTCT | 4-CO1-R | AGGATTACTCCGGTGAGACC | In this study |
| 61 | 4-CO2-F | CTACACCTACACGATCACGC | 4-ATP6-R | TGTGATAGCCAGCGTTGGC | In this study |
| 62 | 4-CO3-F2 | ACGCCGTCTATGGCTCTACA | 4-ND3-R | GGGCGGACTAGATTGAGCAGA | In this study |
| 63 | T-12S-F | CCTGAAGTTGCCGTKATGGA | T-12S-R | CCAAGRGCACTTTCCAGTA | In this study |
| 64 | T-tRNA-Val-F | CCCGTCACCCTCATYAACA | T-16S1-R | CGCTACCTTTGCACGGTTAG | In this study |
| 65 | T-16S2-F | GCCYTMAAGCAGCCACCAA | T-tRNA-Leu-R | GGTGGCAGAGCCAGGTTCTATG | In this study |
