# Supplementary material for: Characterization of five complete Cyrtodactylus mitogenome structures reveals low structural diversity and conservation of repeated sequences in the lineage
Source: PeerJ. 2018 Dec 13;6:e6121. doi: 10.7717/peerj.6121 (PMC6295329; doi:10.7717/peerj.6121)
Supplement: Table S2 [file peerj-06-6121-s004.docx]

**Table S2** Usage of initial and termination codons in five *Cyrtodactylus* species

| Species/Gene | *Cyrtodactylus peguensis* | | *Cyrtodactylus thirakhupti* | | *Cyrtodactylus auribalteatus* | | *Cyrtodactylus chanhomeae* | | *Cyrtodactylus tigroides* | |
| --- | --- | --- | --- | --- | --- | --- | --- | --- | --- | --- |
|  | Start | Stop | Start | Stop | Start | Stop | Start | Stop | Start | Stop |
| *ND1* | ATG | TAG | ATG | AGA | GTG | TAA | GTG | TAA | GTG | TAA |
| *ND2* | ATA | TAA | ATA | TAA | ATA | TAG | ATA | TAG | ATA | TAA |
| *COI* | ATG | AGG | ATG | AGA | ATG | AGA | ATG | AGA | ATG | TAA |
| *COII* | ATG | T++^1^ | ATG | T++^1^ | ATG | T++^1^ | GTG | TAA | ATG | T++^1^ |
| *ATPase8* | ATG | TAA | ATG | TAA | GTG | TAA | ATG | TAA | ATG | TAA |
| *ATPase6* | ATG | TAA | ATG | TAA | ATG | TAA | ATG | TAA | ATG | TAA |
| *COIII* | ATG | T++^1^ | ATG | TA+^1^ | ATG | TA+^1^ | ATG | TA+^1^ | ATG | T++^1^ |
| *ND3* | ATA | TAG | ATA | TA | ATG | TA+^1^ | ATA | TA+^1^ | ATG | TAG |
| *ND4L* | ATG | TAA | ATG | TAA | ATG | TAA | ATG | TAA | ATG | TAA |
| *ND4* | ATG | T++^1^ | ATG | T++^1^ | ATG | T++^1^ | ATG | TAG | ATG | T++^1^ |
| *ND5* | ATG | TAG | ATA | TAG | ATG | TAA | ATG | TAA | ATA | TAA |
| *ND6* | ATG | TAG | ATG | AGG | ATG | TAG | ATG | TAG | ATG | TAG |
| *CYTB* | ATG | TAG | ATG | TAG | ATG | TA+^1^ | ATG | TAA | ATG | TAG |

^1^TAA stop codon is completed by the addition of 3'A residues to mRNA
