# Supplementary material for: Characterization of five complete Cyrtodactylus mitogenome structures reveals low structural diversity and conservation of repeated sequences in the lineage
Source: PeerJ. 2018 Dec 13;6:e6121. doi: 10.7717/peerj.6121 (PMC6295329; doi:10.7717/peerj.6121)
Supplement: Table S3 [file peerj-06-6121-s005.docx]

**Table S3** Mitochondrial genome organization and features in *Cyrtodactylus peguensis*

|  |  | Mitochondrial genome of *Cyrtodactylus peguensis* | | | | | | | Spacer/ overlap |
| --- | --- | --- | --- | --- | --- | --- | --- | --- | --- |
| Gene/element | Strand | Position number | | Size (bp) | Codon | | Codon | Anticodon |  |
|  |  | From | To |  | start | stop |  |  |  |
| tRNA Phe | H | 1 | 73 | 73 |  |  | TTC | GAA |  |
| 12S rRNA | H | 75 | 1020 | 946 |  |  |  |  | 1 |
| tRNA Val | H | 1020 | 1085 | 66 |  |  | GTA | TAC | -1 |
| 16S rRNA | H | 1087 | 2620 | 1534 |  |  |  |  | 1 |
| tRNA Leu | H | 2623 | 2697 | 75 |  |  | TTA | TAA | 2 |
| *ND1* | H | 2698 | 3663 | 966 | ATG | TAG |  |  | 0 |
| tRNA Ile | H | 3666 | 3734 | 69 |  |  | ATC | GAT | 2 |
| tRNA Gln | L | 3733 | 3805 | 73 |  |  | CAA | TTG | -2 |
| tRNA Met | H | 3805 | 3875 | 71 |  |  | ATG | CAT | -1 |
| *ND2* | H | 3876 | 4907 | 1032 | ATA | TAA |  |  | 0 |
| tRNA Trp | H | 4906 | 4973 | 68 |  |  | TGA | TCA | -2 |
| tRNA Ala | L | 4973 | 5038 | 66 |  |  | GCA | TGC | -1 |
| tRNA Asn | L | 5040 | 5112 | 73 |  |  | AAC | GTT | 1 |
| tRNA Cys | L | 5140 | 5197 | 58 |  |  | TGC | GCA | 27 |
| tRNA Tyr | L | 5198 | 5256 | 59 |  |  | TAC | GTA | 0 |
| *COI* | H | 5261 | 6820 | 1560 | ATG | AGG |  |  | 4 |
| tRNA Ser | L | 6809 | 6879 | 71 |  |  | TCA | TGA | -12 |
| tRNA Asp | H | 6880 | 6947 | 68 |  |  | GAC | GTC | 0 |
| *COII* | H | 6948 | 7635 | 688 | ATG | T^2^ |  |  | 0 |
| tRNA Lys | H | 7636 | 7703 | 68 |  |  | AAA | TTT | 0 |
| *ATP8* | H | 7704 | 7865 | 162 | ATG | TAA |  |  | 0 |
| *ATP6* | H | 7856 | 8536 | 681 | ATG | TAA |  |  | -10 |
| *COIII* | H | 8536 | 9319 | 784 | ATG | T^2^ |  |  | -1 |
| tRNA Gly | H | 9320 | 9386 | 67 |  |  | GGA | TCC | 0 |
| *ND3* | H | 9387 | 9743 | 357 | ATA | TAG |  |  | 0 |
| tRNA Arg | H | 9734 | 9804 | 71 |  |  | CGA | TCG | -10 |
| *ND4L* | H | 9807 | 10101 | 295 | ATG | T^2^ |  |  | 2 |
| *ND4* | H | 10098 | 11466 | 1369 | ATG | T^2^ |  |  | -4 |
| tRNA His | H | 11467 | 11535 | 69 |  |  | CAC | GTG | 0 |
| tRNA Ser | H | 11536 | 11595 | 60 |  |  | AGC | GCT | 0 |
| tRNA Leu | H | 11596 | 11667 | 72 |  |  | CTA | TAG | 0 |
| *ND5* | H | 11670 | 13475 | 1806 | ATG | TAG |  |  | 2 |
| *ND6* | L | 13461 | 13985 | 525 | ATG | TAG |  |  | -15 |
| tRNA Glu | L | 13986 | 14056 | 71 |  |  | GAA | TTC | 0 |
| *Cytb* | H | 14060 | 15199 | 1140 | ATG | TAG |  |  | 3 |
| tRNA Thr | H | 15199 | 15270 | 72 |  |  | ACA | TGT | -1 |
| tRNA Pro | L | 15271 | 15335 | 65 |  |  | CCA | TGG | 0 |
| control region |  | 15336 | 16988 | 1653 |  |  |  |  |  |

^1^translation except for position 1..3, amino acid: Met.

^2^TAA stop codon is completed by the addition of 3'A residues to mRNA.
