# Supplementary material for: Characterization of five complete Cyrtodactylus mitogenome structures reveals low structural diversity and conservation of repeated sequences in the lineage
Source: PeerJ. 2018 Dec 13;6:e6121. doi: 10.7717/peerj.6121 (PMC6295329; doi:10.7717/peerj.6121)
Supplement: Table S4 [file peerj-06-6121-s006.docx]

**Table S4** Mitochondrial genome organization and features in *Cyrtodactylus thirakhupti*

|  |  | Mitochondrial genome of *Cyrtodactylus thirakhupti* | | | | | | | Spacer/ Overlap |
| --- | --- | --- | --- | --- | --- | --- | --- | --- | --- |
| Gene/element | Strand | Position number | | Size (bp) | Codon | | Codon | Anticodon |  |
|  |  | From | To |  | start | stop |  |  |  |
| tRNA Phe | H | 1 | 74 | 74 |  |  | TTC | GAA |  |
| 12S rRNA | H | 75 | 1023 | 949 |  |  |  |  | 0 |
| tRNA Val | H | 1023 | 1089 | 67 |  |  | GTA | TAC | -1 |
| 16S rRNA | H | 1090 | 2626 | 1537 |  |  |  |  | 0 |
| tRNA Leu | H | 2629 | 2703 | 75 |  |  | TTA | TAA | 2 |
| *ND1* | H | 2704 | 3669 | 966 | ATG | AGA |  |  | 0 |
| tRNA Ile | H | 3670 | 3739 | 70 |  |  | ATC | GAT | 0 |
| tRNA Gln | L | 3738 | 3810 | 73 |  |  | CAA | TTG | -2 |
| tRNA Met | H | 3810 | 3880 | 71 |  |  | ATG | CAT | -1 |
| *ND2* | H | 3881 | 4912 | 1032 | ATA | TAA |  |  | 0 |
| tRNA Trp | H | 4911 | 4982 | 72 |  |  | TGA | TCA | -2 |
| tRNA Ala | L | 4982 | 5047 | 66 |  |  | GCA | TGC | -1 |
| tRNA Asn | L | 5049 | 5121 | 73 |  |  | AAC | GTT | 1 |
| tRNA Cys | L | 5150 | 5207 | 58 |  |  | TGC | GCA | 28 |
| tRNA Tyr | L | 5208 | 5268 | 61 |  |  | TAC | GTA | 0 |
| *COI* | H | 5273 | 6820 | 1548 | ATG | AGA |  |  | 4 |
| tRNA Ser | L | 6814 | 6884 | 71 |  |  | TCA | TGA | -7 |
| tRNA Asp | H | 6885 | 6952 | 68 |  |  | GAC | GTC | 0 |
| *COII* | H | 6953 | 7640 | 688 | ATG | T^2^ |  |  | 0 |
| tRNA Lys | H | 7641 | 7709 | 69 |  |  | AAA | TTT | 0 |
| *ATP8* | H | 7710 | 7874 | 165 | ATG | TAA |  |  | 0 |
| *ATP6* | H | 7865 | 8545 | 681 | ATG | TAA |  |  | -10 |
| *COIII* | H | 8545 | 9329 | 785 | ATG | TA^2^ |  |  | -1 |
| tRNA Gly | H | 9329 | 9394 | 66 |  |  | GGA | TCC | -1 |
| *ND3* | H | 9395 | 9741 | 347 | ATA | TA^2^ |  |  | 0 |
| tRNA Arg | H | 9742 | 9811 | 70 |  |  | CGA | TCG | 0 |
| *ND4L* | H | 9814 | 10110 | 297 | ATG | TAA |  |  | 2 |
| *ND4* | H | 10104 | 11472 | 1369 | ATG | T^2^ |  |  | -7 |
| tRNA His | H | 11473 | 11542 | 70 |  |  | CAC | GTG | 0 |
| tRNA Ser | H | 11543 | 11600 | 58 |  |  | AGC | GCT | 0 |
| tRNA Leu | H | 11600 | 11671 | 72 |  |  | CTA | TAG | -1 |
| *ND5* | H | 11686 | 13482 | 1797 | ATA | TAG |  |  | 14 |
| *ND6* | L | 13453 | 13989 | 537 | ATG | TAG |  |  | -30 |
| tRNA Glu | L | 13990 | 14060 | 71 |  |  | GAA | TTC | 0 |
| *Cytb* | H | 14064 | 15203 | 1140 | ATG | TAG |  |  | 3 |
| tRNA Thr | H | 15203 | 15273 | 71 |  |  | ACA | TGT | -1 |
| tRNA Pro | L | 15273 | 15341 | 69 |  |  | CCA | TGG | -1 |
| control region |  | 15342 | 16795 | 1454 |  |  |  |  |  |

^1^translation except for position 1..3, amino acid: Met.

^2^TAA stop codon is completed by the addition of 3'A residues to mRNA.
