# Supplementary material for: Characterization of five complete Cyrtodactylus mitogenome structures reveals low structural diversity and conservation of repeated sequences in the lineage
Source: PeerJ. 2018 Dec 13;6:e6121. doi: 10.7717/peerj.6121 (PMC6295329; doi:10.7717/peerj.6121)
Supplement: Table S5 [file peerj-06-6121-s007.docx]

**Table S5** Mitochondrial genome organization and features in *Cyrtodactylus auribalteatus*

|  |  | Mitochondrial genome of *Cyrtodactylus auribalteatus* | | | | | | | Spacer/ Overlap |
| --- | --- | --- | --- | --- | --- | --- | --- | --- | --- |
| Gene/element | Strand | Position number | | Size (bp) | Codon | | Codon | Anticodon |  |
|  |  | From | To |  | start | stop |  |  |  |
| tRNA Phe | H | 1 | 74 | 74 |  |  | TTC | GAA |  |
| 12S rRNA | H | 75 | 1028 | 954 |  |  |  |  | 0 |
| tRNA Val | H | 1028 | 1093 | 66 |  |  | GTA | TAC | -1 |
| 16S rRNA | H | 1094 | 2629 | 1536 |  |  |  |  | 0 |
| tRNA Leu | H | 2632 | 2706 | 75 |  |  | TTA | TAA | 2 |
| *ND1* | H | 2707 | 3675 | 969 | GTG^1^ | TAA |  |  | 0 |
| tRNA Ile | H | 3677 | 3746 | 70 |  |  | ATC | GAT | 1 |
| tRNA Gln | L | 3745 | 3817 | 73 |  |  | CAA | TTG | -2 |
| tRNA Met | H | 3817 | 3886 | 70 |  |  | ATG | CAT | -1 |
| *ND2* | H | 3887 | 4921 | 1035 | ATA | TAG |  |  | 0 |
| tRNA Trp | H | 4920 | 4989 | 70 |  |  | TGA | TCA | -2 |
| tRNA Ala | L | 4989 | 5054 | 66 |  |  | GCA | TGC | -1 |
| tRNA Asn | L | 5056 | 5128 | 73 |  |  | AAC | GTT | 1 |
| tRNA Cys | L | 5157 | 5215 | 59 |  |  | TGC | GCA | 28 |
| tRNA Tyr | L | 5216 | 5274 | 59 |  |  | TAC | GTA | 0 |
| *COI* | H | 5280 | 6827 | 1548 | ATG | TAG |  |  | 5 |
| tRNA Ser | L | 6821 | 6892 | 72 |  |  | TCA | TGA | -7 |
| tRNA Asp | H | 6893 | 6959 | 67 |  |  | GAC | GTC | 0 |
| *COII* | H | 6960 | 7647 | 688 | ATG | T^2^ |  |  | 0 |
| tRNA Lys | H | 7648 | 7715 | 68 |  |  | AAA | TTT | 0 |
| *ATP8* | H | 7716 | 7880 | 165 | GTG^1^ | TAA |  |  | 0 |
| *ATP6* | H | 7871 | 8548 | 678 | ATG | TAA |  |  | -10 |
| *COIII* | H | 8548 | 9329 | 782 | ATG | TA^2^ |  |  | -1 |
| tRNA Gly | H | 9329 | 9394 | 66 |  |  | GGA | TCC | -1 |
| *ND3* | H | 9395 | 9741 | 347 | ATG | TA^2^ |  |  | 0 |
| tRNA Arg | H | 9742 | 9811 | 70 |  |  | CGA | TCG | 0 |
| *ND4L* | H | 9814 | 10110 | 297 | ATG | TAA |  |  | 2 |
| *ND4* | H | 10104 | 11466 | 1363 | ATG | T^2^ |  |  | -7 |
| tRNA His | H | 11476 | 11545 | 70 |  |  | CAC | GTG | 9 |
| tRNA Ser | H | 11546 | 11605 | 60 |  |  | AGC | GCT | 0 |
| tRNA Leu | H | 11606 | 11676 | 71 |  |  | CTA | TAG | 0 |
| *ND5* | H | 11680 | 13488 | 1809 | ATG | TAA |  |  | 3 |
| *ND6* | L | 13474 | 14001 | 528 | ATG | TAG |  |  | -15 |
| tRNA Glu | L | 14002 | 14072 | 71 |  |  | GAA | TTC | 0 |
| *Cytb* | H | 14076 | 15214 | 1139 | ATG | TA^2^ |  |  | 3 |
| tRNA Thr | H | 15215 | 15283 | 69 |  |  | ACA | TGT | 0 |
| tRNA Pro | L | 15284 | 15350 | 67 |  |  | CCA | TGG | 0 |
| control region |  | 15351 | 16795 | 1445 |  |  |  |  |  |

^1^translation except for position 1..3, amino acid: Met.

^2^TAA stop codon is completed by the addition of 3'A residues to mRNA.
