# Supplementary material for: Characterization of five complete Cyrtodactylus mitogenome structures reveals low structural diversity and conservation of repeated sequences in the lineage
Source: PeerJ. 2018 Dec 13;6:e6121. doi: 10.7717/peerj.6121 (PMC6295329; doi:10.7717/peerj.6121)
Supplement: Table S6 [file peerj-06-6121-s008.docx]

**Table S6** Mitochondrial genome organization and features in *Cyrtodactylus chanhomeae*

|  |  | Mitochondrial genome of *Cyrtodactylus auribalteatus* | | | | | | | Spacer/ Overlap |
| --- | --- | --- | --- | --- | --- | --- | --- | --- | --- |
| Gene/element | Strand | Position number | | Size (bp) | Codon | | Codon | Anticodon |  |
|  |  | From | To |  | start | stop |  |  |  |
| tRNA Phe | H | 1 | 72 | 72 |  |  | TTC | GAA |  |
| 12S rRNA | H | 73 | 1030 | 958 |  |  |  |  | 0 |
| tRNA Val | H | 1030 | 1095 | 66 |  |  | GTA | TAC | -1 |
| 16S rRNA | H | 1096 | 2628 | 1533 |  |  |  |  | 0 |
| tRNA Leu | H | 2631 | 2706 | 76 |  |  | TTA | TAA | 2 |
| *ND1* | H | 2707 | 3672 | 966 | GTG^1^ | TAA |  |  | 0 |
| tRNA Ile | H | 3674 | 3743 | 70 |  |  | ATC | GAT | 1 |
| tRNA Gln | L | 3742 | 3813 | 72 |  |  | CAA | TTG | -2 |
| tRNA Met | H | 3813 | 3883 | 71 |  |  | ATG | CAT | -1 |
| *ND2* | H | 3884 | 4921 | 1038 | ATA | TAG |  |  | 0 |
| tRNA Trp | H | 4920 | 4990 | 71 |  |  | TGA | TCA | -2 |
| tRNA Ala | L | 4990 | 5054 | 65 |  |  | GCA | TGC | -1 |
| tRNA Asn | L | 5055 | 5127 | 73 |  |  | AAC | GTT | 0 |
| tRNA Cys | L | 5155 | 5217 | 63 |  |  | TGC | GCA | 27 |
| tRNA Tyr | L | 5218 | 5280 | 63 |  |  | TAC | GTA | 0 |
| *COI* | H | 5284 | 6828 | 1545 | ATG | AGA |  |  | 3 |
| tRNA Ser | L | 6822 | 6894 | 73 |  |  | TCA | TGA | -7 |
| tRNA Asp | H | 6896 | 6963 | 68 |  |  | GAC | GTC | 1 |
| *COII* | H | 6964 | 7638 | 675 | GTG^1^ | TAA |  |  | 0 |
| tRNA Lys | H | 7651 | 7717 | 67 |  |  | AAA | TTT | 12 |
| *ATP8* | H | 7718 | 7885 | 168 | ATG | TAA |  |  | 0 |
| *ATP6* | H | 7876 | 8556 | 681 | ATG | TAA |  |  | -10 |
| *COIII* | H | 8556 | 9340 | 785 | ATG | TA^2^ |  |  | -1 |
| tRNA Gly | H | 9340 | 9404 | 65 |  |  | GGA | TCC | -1 |
| *ND3* | H | 9405 | 9751 | 347 | ATA | TA^2^ |  |  | 0 |
| tRNA Arg | H | 9752 | 9818 | 67 |  |  | CGA | TCG | 0 |
| *ND4L* | H | 9821 | 10117 | 297 | ATG | TAA |  |  | 2 |
| *ND4* | H | 10111 | 11475 | 1365 | ATG | TAG |  |  | -7 |
| tRNA His | H | 11480 | 11549 | 70 |  |  | CAC | GTG | 4 |
| tRNA Ser | H | 11550 | 11608 | 59 |  |  | AGC | GCT | 0 |
| tRNA Leu | H | 11610 | 11681 | 72 |  |  | CTA | TAG | 1 |
| *ND5* | H | 11684 | 13483 | 1800 | ATG | TAA |  |  | 2 |
| *ND6* | L | 13469 | 13993 | 525 | ATG | TAG |  |  | -15 |
| tRNA Glu | L | 13994 | 14062 | 69 |  |  | GAA | TTC | 0 |
| *Cytb* | H | 14065 | 15204 | 1140 | ATG | TAA |  |  | 2 |
| tRNA Thr | H | 15206 | 15275 | 70 |  |  | ACA | TGT | 1 |
| tRNA Pro | L | 15276 | 15340 | 65 |  |  | CCA | TGG | 0 |
| control region |  | 15341 | 17068 | 1728 |  |  |  |  |  |

^1^translation except for position 1..3, amino acid: Met.

^2^TAA stop codon is completed by the addition of 3'A residues to mRNA.
