# Supplementary material for: Characterization of five complete Cyrtodactylus mitogenome structures reveals low structural diversity and conservation of repeated sequences in the lineage
Source: PeerJ. 2018 Dec 13;6:e6121. doi: 10.7717/peerj.6121 (PMC6295329; doi:10.7717/peerj.6121)
Supplement: Table S7 [file peerj-06-6121-s009.docx]

**Table S7** Mitochondrial genome organization and features in *Cyrtodactylus tigroides*

|  |  | Mitochondrial genome of *Cyrtodactylus auribalteatus* | | | | | | | Spacer/ Overlap |
| --- | --- | --- | --- | --- | --- | --- | --- | --- | --- |
| Gene/element | Strand | Position number | | Size (bp) | Codon | | Codon | Anticodon |  |
|  |  | From | To |  | start | stop |  |  |  |
| tRNA Phe | H | 1 | 74 | 74 |  |  | TTC | GAA |  |
| 12S rRNA | H | 75 | 1029 | 955 |  |  |  |  | 0 |
| tRNA Val | H | 1029 | 1094 | 66 |  |  | GTA | TAC | -1 |
| 16S rRNA | H | 1095 | 2639 | 1545 |  |  |  |  | 0 |
| tRNA Leu | H | 2645 | 2720 | 76 |  |  | TTA | TAA | 5 |
| *ND1* | H | 2721 | 3689 | 969 | GTG^1^ | TAA |  |  | 0 |
| tRNA Ile | H | 3691 | 3760 | 70 |  |  | ATC | GAT | 1 |
| tRNA Gln | L | 3759 | 3831 | 73 |  |  | CAA | TTG | -2 |
| tRNA Met | H | 3831 | 3901 | 71 |  |  | ATG | CAT | -1 |
| *ND2* | H | 3902 | 4939 | 1038 | ATA | TAA |  |  | 0 |
| tRNA Trp | H | 4938 | 5008 | 71 |  |  | TGA | TCA | -2 |
| tRNA Ala | L | 5008 | 5073 | 66 |  |  | GCA | TGC | -1 |
| tRNA Asn | L | 5075 | 5147 | 73 |  |  | AAC | GTT | 1 |
| tRNA Cys | L | 5176 | 5233 | 58 |  |  | TGC | GCA | 28 |
| tRNA Tyr | L | 5234 | 5287 | 54 |  |  | TAC | GTA | 0 |
| *COI* | H | 5296 | 6813 | 1518 | ATG | TAA |  |  | 8 |
| tRNA Ser | L | 6842 | 6903 | 62 |  |  | TCA | TGA | 28 |
| tRNA Asp | H | 6918 | 6985 | 68 |  |  | GAC | GTC | 14 |
| *COII* | H | 6986 | 7667 | 682 | ATG | T^2^ |  |  | 0 |
| tRNA Lys | H | 7671 | 7736 | 66 |  |  | AAA | TTT | 3 |
| *ATP8* | H | 7738 | 7902 | 165 | ATG | TAA |  |  | 1 |
| *ATP6* | H | 7893 | 8567 | 675 | ATG | TAA |  |  | -10 |
| *COIII* | H | 8567 | 9350 | 784 | ATG | T^2^ |  |  | -1 |
| tRNA Gly | H | 9351 | 9416 | 66 |  |  | GGA | TCC | 0 |
| *ND3* | H | 9417 | 9773 | 357 | ATG | TAG |  |  | 0 |
| tRNA Arg | H | 9764 | 9833 | 70 |  |  | CGA | TCG | -10 |
| *ND4L* | H | 9837 | 10133 | 297 | ATG | TAA |  |  | 3 |
| *ND4* | H | 10127 | 11489 | 1363 | ATG | T^2^ |  |  | -7 |
| tRNA His | H | 11493 | 11562 | 70 |  |  | CAC | GTG | 3 |
| tRNA Ser | H | 11563 | 11621 | 59 |  |  | AGC | GCT | 0 |
| tRNA Leu | H | 11622 | 11693 | 72 |  |  | CTA | TAG | 0 |
| *ND5* | H | 11708 | 13504 | 1797 | ATG | TAA |  |  | 14 |
| *ND6* | L | 13490 | 14011 | 522 | ATG | TAG |  |  | -15 |
| tRNA Glu | L | 14012 | 14082 | 71 |  |  | GAA | TTC | 0 |
| *Cytb* | H | 14086 | 15231 | 1146 | ATG | TAG |  |  | 3 |
| tRNA Thr | H | 15231 | 15302 | 72 |  |  | ACA | TGT | -1 |
| tRNA Pro | L | 15302 | 15372 | 71 |  |  | CCA | TGG | -1 |
| control region |  | 15373 | 16929 | 1557 |  |  |  |  |  |

^1^translation except for position 1..3, amino acid: Met.

^2^TAA stop codon is completed by the addition of 3'A residues to mRNA.
