# Supplementary material for: Characterization of five complete Cyrtodactylus mitogenome structures reveals low structural diversity and conservation of repeated sequences in the lineage
Source: PeerJ. 2018 Dec 13;6:e6121. doi: 10.7717/peerj.6121 (PMC6295329; doi:10.7717/peerj.6121)
Supplement: Table S8 [file peerj-06-6121-s010.docx]

**Table S8** Variability and similarity between repeated sequences in a non-coding control region among gecko lizards

| Species | GenBank  accession number | VNTR^1^ at ETAS^2^ | | | | VNTR^1^ at CSB^3^ | | | |  |
| --- | --- | --- | --- | --- | --- | --- | --- | --- | --- | --- |
|  |  | Size (bp) | Copy number | Overlap size & similarity | Similarity (%) with 75-bp box (%) | Size  (bp) | Copy number | Overlap size & similarity | Similarity (%) with 75-bp box (%) | |
| *Cyrtodactylus auribalteatus* | AP018116 | 225 | 2 |  |  | 13 | 2 |  |  | |
| *Cyrtodactylus tigroides* | AP018118 | 75 | 6 |  |  | 2 | 34 |  |  | |
| *Cyrtodactylus thirakhupti* | AP018115 | 75 | 6 |  |  | 13 | 3 |  |  | |
|  |  |  |  |  |  | 2 | 19 |  |  | |
| *Cyrtodactylus peguensis* | AP018114 | 75 | 6 |  |  | 43 | 2 |  |  | |
| *Cyrtodactylus chanhomeae* | AP018117 | 75 | 8 |  |  | 2 | 42 |  |  | |
| *Coleonyx variegatus* | NC_008774.1 | 117 | 4 | 97 bp (45.4%) | 58.7% |  |  |  |  | |
| *Goniurosaurus luii* | NC_026105.1 | 91 | 6 | 91 bp (49.0%) | 59.5% |  |  |  |  | |
| *Eublepharis macularius* | NC_033383.1 | 62 | 4 | 52 bp (57.7%) | 40.0% | 30 | 6 | 17 bp (64.7%) | 14.6% | |
|  |  |  |  |  |  | 105 | 6 | 97 bp (53.6%) | 69.3% | |
| *Hemitheconyx caudicinctus* | NC_018368.1 |  |  |  |  | 42 | 4 | 51 bp (47.1%) | 32.0% | |
|  |  |  |  |  |  | 51 | 2 | 60 bp (61.7%) | 49.4% | |
| *Aprasia parapulchella* | NC_024557.1 | 72 | 2 | 41 bp (63.4%) | 34.6% | 10 | 5 | 6 bp (83.3%) | 6.7% | |
| *Cnemaspis limi* | NC_020039.1 | 76 | 4 | 53 bp (69.8%) | 49.3% | 21 | 2 | 8 bp (75.0%) | 8.0% | |
| *Tropiocolotes tripolitanus* | NC_025780.1 | 74 | 4 | 53 bp (86.8%) | 61.3% | 20 | 9 | 12 bp (75.0%) | 12.0% | |
|  |  |  |  |  |  | 100 | 10 | 62 bp (58.1%) | 48.0% | |
| *Stenodactylus petrii* | NC_025784.1 | 74 | 3 | 52 bp (82.7%) | 57.3% | 32 | 3 | 7 bp (85.7%) | 8.0% | |
|  |  |  |  |  |  | 30 | 5 | 26 bp (65.4%) | 22.7% | |
| *Hemidactylus frenatus* | NC_012902.2 | 74 | 5 | 54 bp (83.3%) | 60.0% |  |  |  |  | |
| *Lepidodactylus lugubris* | NC_025782.1 | 75 | 4 | 58 bp (84.5%) | 65.3% | 11 | 12 | 10 bp (80.0%) | 10.7% | |
| *Gekko gecko* | NC_007627.1 | 75 | 2 | 63 bp (77.8%) | 65.4% |  |  |  |  | |
| *Gekko chinensis* | NC_027191.1 | 75 | 5 | 57 bp (84.2%) | 64.0% | 15 | 11 | 12 bp (66.7%) | 10.7% | |
|  |  |  |  |  |  | 114 | 8 | 105 bp (51.4%) | 72.0% | |
| *Gekko japonicus* | NC_028035.1 |  |  |  |  | 67 | 2 | 67 bp (45.7%) | 40.8% | |
| *Gekko swinhonis* | NC_018050.1 | 74 | 6 | 53 bp (86.8%) | 61.3% | 11 | 10 | 7 bp (85.7%) | 8.0% | |
| *Gekko vittatus* | NC_008772.1 | 75 | 2 | 56 bp (89.3%) | 66.7% | 27 | 2 | 12 bp (75.0%) | 12.0% | |
|  |  |  |  |  |  | 11 | 5 | 11 bp (100.0%) | 14.6% | |
| *Uroplatus fimbriatus* | NC_025779.1 |  |  |  |  | 14 | 3 | 11 bp (59.3%) | 8.7% | |
| *Uroplatus ebenaui* | NC_025783.1 |  |  |  |  | 27 | 2 | 18 bp (83.3%) | 20.0% | |
|  |  |  |  |  |  | 11 | 7 | 9 bp (77.8%) | 9.3% | |
|  |  |  |  |  |  | 51 | 4 | 51 bp (42.2%) | 28.7% | |
|  |  |  |  |  |  |  |  |  |  | |
|  |  |  |  |  |  |  |  |  |  | |
|  |  |  |  |  |  |  |  |  |  | |
| *Phelsuma guimbeaui* | AB661664.1 | 75 | 5 | 50 bp (82.0%) | 54.7% | 90 | 2 | 70 bp (54.3%) | 50.7% | |
|  |  |  |  |  |  | 6 | 31 | 5 bp (80.0%) | 5.3% | |
|  |  |  |  |  |  | 44 | 19 | 44 bp (55.8%) | 32.7% | |
| *Paroedura picta* | NC_028326.1 | 74 | 4 | 63 bp (77.8%) | 65.4% | 59 | 3 | 59 bp (38.7%) | 30.4% | |
| *Phyllodactylus unctus* | NC_020038.1 | 75 | 4 | 67 bp (88.1%) | 78.7% | 12 | 2 | 4 bp (100.0%) | 5.3% | |
|  |  |  |  |  |  | 6 | 5 | 5 bp (80.0%) | 5.3% | |
|  |  |  |  |  |  | 6 | 35 | 5 bp (100.0%) | 6.7% | |
|  |  |  |  |  |  | 11 | 8 | 10 bp (90.9%) | 12.1% | |
| *Tarentola mauritanica* | NC_012366.1 | 75 | 6 | 68 bp (86.8%) | 78.7% |  |  |  |  | |
|  |  | 66 | 11 | 66 bp (47.4%) | 41.7% |  |  |  |  | |
| *Heteronotia binoei* | NC_010292.1 | 74 | 3 | 59 bp (78.0%) | 61.4% | 11 | 4 | 10 bp (80.0%) | 10.7% | |
|  |  | 14 | 2 | 9 bp (88.9%) | 10.7% | 67 | 5 | 67 bp (48.6%) | 43.4% | |
|  |  |  |  |  |  | 14 | 9 | 14 bp (75.0%) | 14.0% | |
|  |  |  |  |  |  | 31 | 2 | 31 bp (48.6%) | 20.1% | |
| *Teratoscincus keyserlingii* | AY753545.1 | 48 | 7 | 32 bp (53.1%) | 22.7% | 15 | 6 | 6 bp (83.3%) | 6.7% | |
|  |  |  |  |  |  | 266 | 2 | 111 bp (53.2%) | 78.7% | |
| *Teratoscincus roborowskii* | KP115216.1 | 48 | 2 | 48 bp (49.0%) | 31.4% | 2 | 27 | 2 bp (100.0%) | 2.7% | |
|  |  | 60 | 2 | 39 bp (48.7%) | 25.3% |  |  |  |  | |
| *Hemidactylus bowringii* | NC_025938.1 | 49 | 2 | 34 bp (58.8%) | 26.7% | 2 | 44 | 2 bp (100.0%) | 2.7% | |
|  |  | 75 | 5 | 69 bp (87.0%) | 80.4% |  |  |  |  | |

^1^VNTR indicates variable number of tandem repeat.

^2^ETAS indicates extended termination associated sequence domain.

^3^CSB indicates conserved sequence block domain.
